# Validation of an agent-based model for cell interactions in a microfluidic chip

**DOI:** 10.1371/journal.pone.0341962

**Published:** 2026-02-09

**Authors:** Simona Panunzi, Marcello Pompa, Pietro Marco D’Angelo, Gabriella Bretti, Andrea De Gaetano

**Affiliations:** 1 Institute of Systems Analysis and Informatics “A. Ruberti” (IASI) – National Research Council of Italy, Rome, Italy; 2 Campus Biomedico di Roma, Rome, Italy; 3 Institute for Applied Mathematics “Mauro Picone” (IAC) – National Research Council of Italy, Rome, Italy; 4 Institute for Biomedical Research and Innovation (IRIB) – National Research Council of Italy, Palermo, Italy; 5 Physiological Controls Research Center, Obuda University, Budapest, Hungary; KIST: Korea Institute of Science and Technology, GERMANY

## Abstract

**Objectives:** Microfluidic cell Co-Culture, Tissue Co-Culture and Organ-on-Chip (OoC) technologies enable modeling of tissues and organs in vitro, facilitating cell-environment interaction studies and early therapeutic evaluation. The combination of physiology-based models, agent-based models (ABMs), cellular automata, and in-vitro modelling of complex processes provides a powerful tool to formalize, quantify, and predict observed phenomena.

**Methods:** Estimating parameters for these hybrid computational models using observational data is challenging. Approximate Bayesian computation (ABC) is particularly well suited for this task due to the intractability of the likelihood function. This work extends a hybrid ABM for a cell co-culture experiment on a chip. Cell tracking data is used to estimate model parameters via a Sequential Monte Carlo ABC (ABC-SMC) approach.

**Results:** The resulting model accurately reproduces observed cellular behavior and distinguishes between different experimental conditions.

**Conclusion:** The combination of cell co-culture and microfluidic technology with hybrid computational models and ABC-SMC provides a robust framework for modeling and predicting cellular behavior in vitro, enhancing the potential for early therapeutic evaluation and understanding of cell-environment interactions.

## Introduction

The integration of cell/tissue cultures with microfluidics has led to the development of Microfluidic cell Co-Cultures, Tissue Co-Cultures and Organs-on-Chips (OoCs) [1,2], enabling in vitro simulation of tissue and organ microenvironments. These platforms facilitate studying cell-environment interactions across varying levels of complexity, reproduce physicochemical features observed in vivo to varying degrees, and enable assessing therapeutic efficacy/toxicity [3–7]. Combining physiologically based modeling, ABMs [8–10], cellular automata [11,12], and microfluidic platforms provides a powerful tool for formalizing, quantifying, and forecasting biological phenomena. Cellular automata can represent chip architecture in 2D/3D, with cells characterized by states (e.g., cell identity, chemical concentrations) that change as a function of time and the state of their neighbors. Conversely, Agent-Based Models (ABMs) allow the description of the movement and interactions of different cells moving across the background (represented by the cellular automaton) according to physiologically motivated rules. Within each agent, the dynamics can be represented by systems of differential equations, with parameters specific to the agent, typically extracted from distributions representing the agent population. Hybrid computational models offer flexibility and accuracy, avoiding challenges associated with pure mathematical modeling. However, parameter estimation for these complex models is challenging. Classical statistical methods are often unsuitable due to intractable likelihood functions.

Approximate Bayesian Computation (ABC) bypasses exact likelihood calculations by using simulation-based procedures and summary statistics to represent the most relevant information concisely [13–15]. These methods involve simulating the model multiple times with parameter values sampled from a probability distribution. The simulated data are then reduced to summary statistics, which are compared to those derived from observed data. Sampled parameters are accepted or rejected based on the distance between simulated and observed summary statistics. The accepted values form a sample from the approximate posterior distribution, providing both parameter estimates and a quantification of uncertainty and parameter correlations.

The goal of this work is to validate a modified version of a hybrid mathematical formulation, based on a cellular automaton and agent-based model, recently proposed in [16] for representing a microfluidic-based co-culture experiment [17,18] where doxorubicin (DOXO)-pretreated human MDA-MB-231 breast cancer cells and peripheral blood mononucleated cells (PBMCs) were cultured [19]. The objective of the original biological experiment was to demonstrate that only blood cells expressing the formyl peptide receptor 1 (FPR1) with the CC genotype (homozygosity) were able to activate an anticancer immune response, thanks to their ability to recognize and bind to annexin A1, contrary to blood cells with the heterozygous receptor (FPR1 with CA genotype). Video-microscopy allowed the observation of mononuclear blood cell migration towards cancer cells and their interaction.

The ABM model used here aims to reproduce the observed behavior. We used cell-tracking to quantify cell movement from microphotographs captured every 2 minutes from the original video. The output of the cell-tracking procedure was then used to estimate the ABM model parameters via a Sequential Monte Carlo Approximate Bayesian Computation (SMC-ABC) approach.

Original data corresponding to two original video clips (S13 with homozygotic FPR1 and S16 with heterozygotic FPR1 in the Supplementary Material of Vacchelli et al. [19]) are analyzed here with the same model, and the corresponding parameter estimates are compared.

## Materials and methods

### Experimental setting

**Fig 1:**
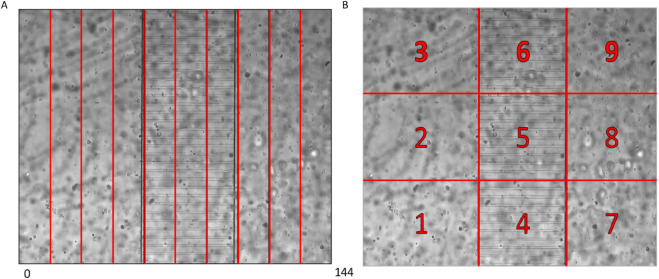
Chip domain. Panel A. Subdivision of the domain chip into 10 bins for the computation of the “Bins” statistic. Panel B. Subdivision of the domain chip into 9 quadrants for the computation of the “Quadrants” statistic.

The microfluidic-based device [17] used for the original biological experiment comprises six reservoirs (diameter 8 mm) for media and cell loading connected to two main culture chambers, each 1mm wide, 8mm long, and 100μm high. These chambers are separated by two central end-closed channels serving as *buffers*, each 4mm in length and 1mm wide. Communication between the main chambers and the central buffer channels occurs via four sets of microchannels (microgrooves), each 12μm wide, 500μm long, and 10μm high. The arrangement of these channels is explicitly designed to mimic physiological cell migration environments such as venules. The microfluidic architecture enables precise spatial separation and staged interaction between two distinct cell populations (e.g., immune cells vs. tumor cells), as well as real-time, high-resolution microscopy. The presence of microchannels permits not only chemical but also physical interaction, making it possible to recapitulate key features of in vivo cell migration, cell-cell contact, and paracrine signaling dynamics. Fig. 1 in S1 Text shows the the microfluidic platform (panel A) and a schematic representation of the two-dimensional planimetry of the central part of the device (panel B). The figure outlines the two central end-closed channels, adjacent to two cell culture compartments, connected via four sets of micron-size channels.

In a subset of experiments, the left reservoirs were cultured with Doxorubicin (DOXO)-pretreated (25μm, 4 hours) human MDA-MB-231 breast cancer cells; the right reservoirs were populated with peripheral blood mononucleated cells (PBMCs) expressing the formyl peptide receptor 1 (FPR1) single nucleotide polymorphism (SNP) rs867228 with CC genotype (referred as “Experiment CC” in the present work) or with CA genotype (“Experiment CA”), a pathogen recognition receptor of ligands such as Annexin A1 (ANXA1). The experiments performed in this microfluidic device aimed to highlight the importance of FPR1 (in homozygosis *FPR*1^*CC*^) in chemotherapy-induced anticancer immune response, determined by its link with Annexin A1.

Before the cells were loaded into the microfluidic device, they were cultured for 24 to 72 hours.

Leukocytes appeared in the right compartment over time through passive migration from the reservoirs. This process occurred progressively during the first 24 hours of the experiment and was not filmed. The cells were then monitored by fluorescence videomicroscopy for further 24 hours and the microphotographs were captured every 2 minutes for a total of 720 frames. During this period leukocytes progressively migrated through the microchannels and reached the tumor cells in the left chamber, following a chemoattractant gradient that was established over time. Additional experiment details are in Vacchelli *et.al* [19]. In what is henceforth called “Experiment CC”, the migration of mononucleated blood cells towards dying cancer cells, as well as their prolonged interaction, were observed when FPR1 was in homozygosis *FPR*1^*CC*^. Conversely, in “Experiment CA” reduced movement and interaction were observed when FPR1 was in heterozygosis *FPR*1^*CA*^. Videos were provided as Supplementary Material of that article [19]. Recordings were performed using a Juli Smart microscope (Digital Bio), and microphotographs were taken only for a defined region of the device (see Fig 1 in S1 Text, panel B). Fig 1 shows a screenshot of one of the two videos.

The mathematical model aims to reproduce the observed behaviour by estimating model parameters from cell tracking data. Additionally, a comparison between results from the estimation procedures on data tracked from “Experiment CC” (movie S13) and “Experiment CA” (movie S16) was performed. Both videos are available as Supplementary Material in [19] at: https://www.science.org/doi/suppl/10.1126/science.aad0779/suppl_file/aad0779-moviess1tos24.zip.

### Computational representation of the microchip domain

The recorded physical domain of the microchip was represented as a bidimensional matrix *M* (Fig 1 in S1 Text, panel B) composed of three sections: two lateral chambers (part of the left main compartment and part of the one central end-closed channel) and a microchannel space in the middle. The component elements of the matrix *M* are squares of size Δx=Δy=12μm, while the total length and height of the domain are $L_{x}=1707\mu m$ and $L_{y}=1452\mu m$, respectively [17]. The width of the left chamber was 676μm, the center width was 500μm, while the right chamber measured 531μm in width. The spatial discretization thus produced, rounding up the result of dividing each width by the discretization size, 57, 42 and 45 columns, respectively, for total of *N*_*col*_ = 144 columns. The number of rows was *N*_*row*_ = 121, determined by the size of the microchannels and the interspace between each pair of microchannels. Since in the bidimensional representation the height of a microchannel is 12μm (the same as the chosen size of the matrix grid square), and the interspace size is 33μm, with a total of 31 channels and 30 interspaces, the total number of rows is given by 12μm/12μm × 31  +  ceil(33μm/12μm)×30=121.

For the domain discretization, each grid square is represented by p=(i,j), where *i* is the *i*–*th* row and *j* is the *j*–*th* column, with *i* = 1..*N*_*row*_ and *j* = 1,...,*N*_*col*_. Moreover, each grid square *p* can be also represented by the coordinates $x_{p}\in L_{x}$ and $y_{p}\in L_{y}$ of center point of the grid square.

Each point of the domain has been initialized to take into account the presence of obstacles, interspace between microchannes, conditioning the movement of leukocytes. To do this we introduce an occupancy labeling function *O*(*x*, *y*) for each grid square in the computational domain $\left(x,y)\in [0,L_{x}]\times [0,L_{y}\right]$ in such a way that:

- O(x,y)=−1 if the point (*x*, *y*) falls on the interspace between two consecutive microchannels;
- O(x,y)=0 otherwise, i.e., if the point (*x*, *y*) is free from obstacles.

The radius of a cancer cell was set to $r_{c}=10\mu m$ while that of a mononuclear cell or leukocyte to $r_{l}=4\mu m$. This means that each grid square inside the microchannel can host only a single leukocyte. The discretization time used for performing the simulations was set to Δt=4min for a total of time steps $N_{t}=\frac{2880min}{4min}=720$ (2880min being 48h). It should be noted that while the video has a duration of 24h, the experiment, and therefore also the simulation, starts 24h beforehand. Julia v1.8.3 was used to implement the model. The simulations were parallelized on a 48-core CPU (2x AMD EPYC 74F3 24-core CPU @ 3.19 GHz) using the “Distributed” package of Julia [20].

### Mathematical modelling

For a detailed description of the model we refer to previous works [16,21]. However, for completeness, the main equations characterizing the model behaviour are listed below. Given the leukocyte *L*, its generic position at time *t*, $\left[X_{L}(t),Y_{L}(t)\right]$, is a function of the parameter θ given by:

θ (1)

where *N*_*L*_(*t*) is the total number of leukocytes at time t∈[0,T]. The function *f*, which defines the agent-based model’s rules, formulas, and equations, depends on *A*(*x*, *y*, *t*) (the space- and time-varying concentration of annexin). This concentration, in turn, is influenced by the number of the cancer cells (*N*_*C*_(*x*,*y*,*t*)) at the position (*x*, *y*) and time *t*, as well as by the parameter vector θ.

Each leukocyte appears randomly, according to a normalized rate *k*_*l*_*eu*1 (see Table 1), in the rightmost half of the right chamber. The grid square it occupies at the beginning of the simulation is chosen randomly from the possible squares in this part of the domain. Moreover, each leukocyte is born with an age that is randomly drawn from a uniform distribution in (0,*L*_*L*_), where *L*_*L*_ is the maximum leukocyte lifetime (Table 1 in S1 Text). At each temporal step the leukocyte age is increased by Δt.

**Table 1:** Free model parameters.

Parameter	Description	Units
*k*_*leu*1_	normalized rate of new leukocyte accrual in the right chamber	$min^{-1}$
*γ*	threshold value for migration	#
*λ*	tendency of a leukocyte to migrate towards higher annexin concentrations	#
*k*_*TL*_	tumor cells life reducing by leukocytes	min
*k*_*dis*_	portion of tumor cells distributed in the lower half of the left chamber	#
*k*_*leu*2_	normalized rate of new leukocyte accrual in the left chamber	$min^{-1}$
*t*_*dly*_	mean delay time for the appearance of a leukocyte in the left chamber	*min*

The movement of the *L*-th leukocyte at time *t*  +  Δt is influenced by its capacity to find its attractor, the annexin, at the previous time *t*.

Given the domain discretization, each *L*–*th* leukocyte possesses, at each time t, the grid square’s position as an attribute.

Let $p_{n}\in N(p)=\{p_{1},\ldots ,p_{N_{p}}\}$ denote the possible neighbor grid squares (according to the Moore neighborhood configuration) where the leukocyte can move (excluding the grid square it occupies at time *t*) and let $\overset{―}{N}(p)=N(p)$ ∪ {*p*} be the “extended” neighborhood of *p* which includes the grid square the leukocyte occupies at time *t*.

The leukocyte tends to move according to an isotropic random walk if the local chemoattractant concentrations are low. The probability of an isotropic random walk is given by

$$P_{MT}=e^{-\gamma T_{A}(\lambda ,t)},$$ (2)

where

$$T_{A}(\lambda ,t)=\sum_{q\in \overset{―}{N}(p)}A(q,t{)}^{\lambda }$$ (3)

denotes the *λ*-adjusted total concentration of annexin in the neighborhood, defined as the sum of the concentrations of the attractor *A*(*q*, *t*) in each neighbour grid square *q* of *p* (including itself). The parameter *γ* represents the sensitivity to the annexin: the lower its value, the higher the probability of an isotropic random walk. The coefficient *λ* expresses the tendency of an agent to migrate towards higher attractor concentrations: the higher the coefficient *λ*, the stronger the preference for moving towards positions at higher attractor concentration.

In the absence of a significant driving gradient, the probability of occupying any neighboring grid square, including its own, is the same and computed as $P_{MT}\frac{1}{N_{p}+1}$. Conversely, when the local annexin concentrations are consistent, the probability of moving towards any grid square in the “extended” neighborhood $\overset{―}{N}(p)$ is proportional to the *λ*-adjusted chemoattractant concentration in that grid square. Therefore, the probability of moving towards a given grid square $q\in \overset{―}{N}(p)$ is

$$P(q,t+\Delta t)=P_{MT}\frac{1}{N_{p}+1}+(1-P_{MT})\frac{A(q,t{)}^{\lambda }}{T_{A}(\lambda ,t)},$$ (4)

from which, by the definition of $T_{A}(\lambda ,t)$ in (3), it follows that $\sum_{q\in \overset{―}{N}(p)}P(q,t+\Delta t)=1\;\;\forall p,\forall t$, given that $|\overset{―}{N}(p)|=N_{p}+1$.

The dynamics of the annexin concentration is described by the following equation:

$$\frac{\partial A(x,y,t)}{\partial t}=k_{A}N_{C}(x,y,t)+D△A(x,y,t)-k_{XA}A(x,y,t),$$ (5)

A(x,y,0)=0∀(x,y)∈Ω

Eq 5 includes the production term *k*_*A*_, the annexin diffusion D△A(x,y,t) and its elimination *k*_*XA*_*A*(*x*,*y*,*t*) in correspondence of (x,y,t)∈Ω×[0,T].

For computational purpose, given the discretized domain, if, as above, *p* represents the generic grid square, *A*(*p*, *t*) is the concentration of annexin at *p* and A(p,t+Δt) can be computed as:


$$A(p,t+\Delta t)=A(p,t)+k_{A}\Delta t-k_{XA}A(p,t)\Delta t+DA_{\text{diff}}(p,t),$$

with $A_{\text{diff}}(p,t)=A(p,t)-\sum_{n=1}^{N_{p}}\frac{A(p,t)}{d_{n}}$ being the concentration of annexin obtained after the sharing among the *N*_*p*_ neighbours of *p*, with $d_{n}=|p-p_{n}|$ being the Euclidean distance among *p* and its generic neighbour *p*_*n*_. As a consequence, at t+Δt the neighbour $p_{n}\in N(p)$ receives part of the amount of annexin shared by *p*:


$$A(p_{n},t+\Delta t)=A(p_{n},t)+\frac{A(p,t)}{d_{n}}.$$

Note that the production in a grid square not occupied by a tumor cell is zero, while the elimination and diffusion of annexin in the environment occurs in each grid square of the domain.

A peculiar feature of the model consists in the description of the interaction between leukocytes and cancer cells: when a leukocyte reaches a tumor cell, its effect on it is that of increasing the biological age of the cell, hence hastening its death (at its predetermined maximum lifetime *L*_*c*_). A tumor cell is deemed “reached by a leukocyte” when the leukocyte, following its movement rules, occupies the same grid square. Furthermore, within the same time step, a tumor cell can be reached by more than one leukocyte and can encounter the same leukocyte multiple times during the simulation. Older cells are more susceptible to leukocyte action:

$$age_{c}:=age_{c}+k_{TL}\left(\frac{age_{c}}{L_{c}}+1\right)$$ (6)

where *k*_*TL*_ represents the intensity of this interaction. The variable *age*_*c*_ was initialized at $age_{c}(t=0)=0\;min$ and, independent of any possible interaction with leukocytes, it is increased by $\Delta_{t}$ at each temporal step.

The symbol “:=” in the above equation denotes an assignment operation in an algorithmic sense, meaning that the variable *age*_*c*_ is updated following Eq 6 when the event “a leukocyte contacts a tumor cell” occurs.

### Model innovations

In this work some changes have been made compared to the formulations in [16] and [21]. While in the original modelling approach the cancer cells were deployed only at the bottom of the left chamber of the considered domain, in this version a new parameter (*k*_*dis*_) allows a random distribution of the total amount of the cancer cells both in the upper and lower zone of the left chamber. In fact, while the original video shows that the majority of cancer cells are located in the lower part of the left chamber (where the interaction with the leukocytes is more evident), nevertheless a fraction of cells is also found in the upper part. This small fraction acts as attractor for a small number of leucocytes.

From visual inspection of the video and from the results of cell tracing it is evident that, after a certain time from the beginning of the experiment, a small number of leukocytes enter the domain from the upper portion of the left compartment. As already mentioned above, the mathematical representation of the chip is related only to that part of the chip for which micro-cinematography is available. The observed leukocytes that arrive from above are therefore those leukocytes that come from the upper part of the right chamber (not represented in the domain considered), and that follow a downward trajectory towards the left. This type of experimental observation cannot be reproduced with the mathematical formalization adopted in either [16] or [21]. In the present work, therefore, it was necessary to introduce some changes. In analogy with the original formulation where the leukocyte accrual rate into the right compartment was represented by the free parameter *k*_*leu*1_ (see Table 1), similarly, in this formalization the new parameter *k*_*leu*2_ has been introduced to represent the accrual rate of leukocytes in the upper part of the left chamber. An additional parameter, *t*_*dly*_ was also introduced to represent the delay time for the appearance of a leukocyte in the left compartment. The introduction of the parameter *k*_*leu*1_ is motivated by the need to maintain computational sustainability for the simulation. Since only the portion of the domain visible in the experimental movie is modeled, the right chamber in the simulation representation is not connected to any physical structure on its right side, as nothing was represented beyond the experimental field of view. To account for the entry of leukocytes into this chamber, the parameter *k*_*leu*1_, which represents the probability that a leukocyte will be spawned at each time step, was therefore introduced. Similarly, the parameter *k*_*leu*2_ represents the probability that, at time *t*_*dly*_, leukocytes will enter from the upper boundary of the left chamber.

Table 1 reports the free model parameters, along with their description, 1 whereas Table 1 in S1 Text reports, for completeness, all the model parameters.

### Leukocyte tracking

For the tracking of leukocyte trajectories, the Python Trackpy library (version 0.5.0) was used. Trackpy is a package for tracking blob-like features in video images, tracing their movements over time, and analysing their trajectories. It employs bandpass filtering and thresholding techniques to identify and track particles within video sequences [22]. To identify leukocytes within the considered video frames, we utilized the ‘trackpy.batch’ function, which systematically detects and records particle positions within each frame on the video and collects the results into a data-frame. This function finds features by determining local maxima of intensity, thereby assuming the particles that need to be tracked have a Gaussian intensity distribution with a maximum in the centre. To distinguish leukocytes from undesired artefacts such as soil impurities, dirt, and air bubbles, we customized several key parameters of the batch function. These modifications included adjustment to parameters expressing the contrast, defining the minimum integrated brightness, representing the average pixel-based diameter and referring to maximum radius-of-gyration of brightness. The specific values chosen for the parameters were guided by empirical observations and prior knowledge, ensuring accurate leukocyte identification. The ‘trackpy.link’ function was used to connect particles across consecutive frames, thus forming trajectories for individual leukocytes. This function enables the seamless tracking of particles by associating them across frames. Key parameters of interest included the maximum allowable distance for features to move between frames, and the number of frames retained for a particle’s identity if it momentarily disappears and reappears within the video sequence.

### Approximate Bayesian computation for model parameter estimation

Approximate Bayesian computation (ABC) is a computational methods used in Bayesian statistics for the estimation of the posterior distributions of model parameters, especially when the likelihood function is difficult to calculate directly or analytically intractable. The approach involves running the model a large number of times with different parameter values, drawn from a pre-specified (proposal) prior distribution. The outcomes of the system simulations are compared with observed data according to some chosen criteria (measuring the level of discrepancy between what is simulated and what has been observed): only the parameter values that produce simulations “close” to the observed data are retained. The sample of parameter values satisfying the criteria determines an approximate empirical parameter posterior distribution. In this way, the explicit calculation of the likelihood function, necessary for a rigorous application of Bayes’ theorem, is avoided. Using this approach, it is possible to obtain both a point estimate of the model’s free parameter values and an estimate of their variability as well as the correlations among parameters.

For the execution of the ABC method, it is therefore necessary to specify the criterion of closeness of the model output, for a given parameter value, to the data. For any (observed or simulated) sample, we must define a statistic capturing the meaningful features of the dataset. For each parameter vector θ generated from the proposal distribution, we then require that the difference between the statistic on the simulated data and the statistic on the observed data (henceforth the difference between simulated and observed statistics) be small, typically smaller than some threshold value set by the user. In the particular case of the present investigation, we defined two different such statistics: one based on the frequency distribution of the leukocytes along the horizontal size of the domain (“Bins” method, Fig 1, panel A) and the other based on the number of leukocytes inside large quadrants that the entire domain was divided into (“Quadrants” method, Fig 1, panel B).

According to the first criterion, for each simulation *i* (corresponding to a parameter vector $\theta_{i}$) we can compute at the time frame *t* the distance $\ell oss_{B_{i_{r}}}^{t}(\theta_{i})$ between the simulation output and the observed data. Note that for each *i*, because the mathematical formulation includes elements of randomness, each simulation represents only one possible realization of the system (the subscript *r* denotes the particular realization). Since we perform only one realization for each parameter set $\theta_{i}$, the subscript *r* can be omitted.

The following equation reports the computation of the criterion in correspondence of the time frame *t*:

$$\ell oss_{B_{i}}^{t}(\theta_{i})=\sum_{j=1}^{J=10}|f_{ij}^{s}(t,\theta_{i})-f_{j}^{o}(t)|\omega_{j}$$ (7)

where

- $f_{ij}^{s}(t,\theta_{i})$ represents the frequency of leukocytes from the realization of the *i*–*th* simulation at time frame *t*, computed for the *j*-th bin, where j=1,…,J denotes the number of intervals into which the horizontal domain has been divided;
- $f_{j}^{o}(t)$ represents the observed absolute frequency of leukocytes at time frame *t* for the *j*-th bin;
- $\omega_{j}$ represents the weight associated with the *j*–*th* deviation, corresponding to the *j*-th bin. The weights are set to 1 for bins 2 to *J*. The first bin (corresponding to the leftmost columns of the domain) was weighted five times more than the others to force leukocytes to reach the farthest portion of the domain. This was necessary because most leukocytes originate from the rightmost side of the domain and must reach the leftmost tumour cells. However, by chance, all the cells could be positioned (since their initial position within the domain is random and unobserved) far from the domain boundary (corresponding to the leftmost columns), preventing leukocytes from reaching the very last portion of the chamber.

The total distance $Loss_{B_{i}}(\theta_{i})$ for the simulation *i* is given by the sum of the loss values computed at chosen time frames, in this case every 40 frames for a total of T=19 frames.

$$Loss_{B_{i}}(\theta_{i})=\sum_{t=1}^{T}\ell oss_{B_{i}}^{t}(\theta_{i})$$ (8)

The second criterion subdivides the chip domain into nine quadrants and counts the number of leukocytes within each. For the simulation *i*, we compute the distance $\ell oss_{Q_{i}}^{t}(\theta_{i})$ between the simulation output and the observed data at time frame *t* as follows:

$$\ell oss_{Q_{i}}^{t}(\theta_{i})=\sum_{k=1}^{K=9}|n_{ik}^{s}(t,\theta_{i})-n_{k}^{o}(t)|$$ (9)

where

- $n_{ik}^{s}(t,\theta_{i})$ represents the number of leukocytes from the i-th simulation at the time frame *t*, computed for the *k*-th quadrant, where k=1,…,K and *K* = 9 is the number of quadrants;
- $n_{k}^{o}(t)$ represents the observed number of leukocytes at time frame *t* computed for the *k*-th quadrant.

The total distance $Loss_{Q_{i}}(\theta_{i})$ for the simulation *i* is given by:

$$Loss_{Q_{i}}(\theta_{i})=\sum_{t=1}^{T}\ell oss_{Q_{i}}^{t}(\theta_{i})$$ (10)

### The sequential Monte Carlo (SMC) ABC method (ABC-SMC)

In the ABC-SMC method [23], a set of randomly chosen parameter values, called particles, $\theta_{1}$,., $\theta_{N_{SMC}}$, are drawn from a starting distribution π(θ). These particles are propagated through a series of intermediate distributions, $\pi (\theta ,d(x_{0},x^{*})<\epsilon_{p})$, p=1,.,P − 1 until the particles are a sample from the target distribution $\pi (\theta ,d(x_{0},x^{*})<\epsilon_{P})$. The tolerances $\epsilon_{i}$ satisfy the condition that $\epsilon_{1}>\epsilon_{2}>...\epsilon_{p}>...\epsilon_{P}$, thus the distributions gradually evolve towards the final posterior distribution. Below the step by step algorithm is reported:

**ABC-SMC Algorithm**

let’s define *Loss*_*M*_, where *M* is one of the two possible methods (*B* for bins and *Q* for quadrants), as the algorithm must be executed once for each method.

choose $\epsilon_{1}$,...,$\epsilon_{p}$,...,$\epsilon_{P}$

set the number of particles *N*_*SMC*_

initialize the particle indicator *i* = 1;

for (p = 1 to P)

  i = 1

  while (i ≤ *N*_*SMC*_)

    set the control parameter *p*_*exit*_ = FALSE

    while (*p*_*exit*_ =  = *FALSE*)

     if (p == 1)

       • sample $\theta^{**}$ randomly from the chosen a-priori distribution π(θ)

     else

       • sample $\theta^{*}$ from the previous population $\left\{\theta_{p-1}^{\left(i\right)}\right\}$ with weights $\left\{w_{p-1}^{\left(i\right)}\right\}$

       • perturb the particle to obtain $\theta^{**}\sim Ke_{p}(\theta |\theta^{*})$, where *Ke*_*p*_ is a perturbation kernel

     end

     if π(θ**) is not 0

       • *p*_*exit*_=TRUE;

     end

    end

    Simulate a realization of the system x^*^ with $\theta^{**}$

    compute the distance $Loss_{M}(\theta^{**})$ (Eq 8 for *B* and Eq 10 for *Q*)

    if $Loss_{M}(\theta^{**})\leq \epsilon_{p}$

       Set $\theta_{p}^{\left(i\right)}=\theta^{**}$, $Loss_{M,p}^{\left(i\right)}=Loss_{M}(\theta^{**})$ and calculate the weight for the particle $\theta_{p}^{\left(i\right)}:$


$$\omega_{p}^{\left(i\right)}=\{\text{1, if}\;\;p=1\;\frac{\pi (\theta_{p}^{\left(i\right)})}{\sum_{g=1}^{N_{SMC}}\omega_{p-1}^{\left(g\right)}Ke_{p}(\theta_{p}^{\left(i\right)}|\theta_{p-1}^{\left(g\right)})},\;\;\text{if}\;\;p>1$$

emsp;   end

     i = i + 1

  end

  Normalize the weights.

end

The denominator in the formula for the computation of the unnormalized weights $\omega_{p}^{\left(i\right)}$ for *p*>1 represents the Monte Carlo approximation of the generic proposal distribution $\eta_{t}(x_{t})$ used for the computation of the importance weights $\omega_{t}(x_{t})=\pi_{t}(x_{t})/\eta_{t}(x_{t})$ in the Sequential Importance Sampling (SIS) algorithm [24]. If $\pi_{T}$ is the target distribution in SIS, $\pi_{t}$ (t=1,...,T − 1) is one of the intermediate distribution (see S1 File for a description of the SIS algorithm). When sampling from $\pi_{t}$ is difficult, leveraging on the idea of the Importance Sampling a series of proposal distributions $\eta_{t}(x_{t})$ can be used and the importance weights $\omega_{t}(x_{t})$ are computed as ratio between $\pi_{t}$ and $\eta_{t}(x_{t})$. In SIS the proposal distributions $\eta_{t}(x_{t})$ are defined as:

$$\eta_{t}(x_{t})=\int \eta_{t-1}(x_{t-1})\kappa_{t}(x_{t-1},x_{t})dx_{t-1}$$ (11)

where $\eta_{t-1}$ is the previous proposal distribution and $\kappa_{t}$ is a Markov kernel.

While a full derivation of $\eta_{t}$ within the ABC-SMC algorithm is outside the scope of the current work, a comprehensive explanation can be found in “APPENDIX A. DERIVATION OF ABC SMC” of [23].

Tolerance values were selected based on the following rationale: for each bin or quadrant in every frame, a positive or negative percentage deviation between predicted and observed frequencies was established to enable the calculation of target values for $Loss_{B_{i}}$ and $Loss_{Q_{i}}$. Percentages 80%, 70%, 60% and 50% were used to determine the sequence of thresholds $\epsilon_{p}$. For both the “Bins” and “Quadrants” methods, this resulted in $\epsilon_{1}$=2303, $\epsilon_{2}$= 2015, $\epsilon_{3}$=1727 and $\epsilon_{4}$=1439. Additionally, the “Quadrants” method considered an extra threshold, $\epsilon_{5}=1200$, corresponding to a deviation of about 40%.

A Gaussian kernel was used as perturbation kernel $Ke_{p}(\theta |\theta^{*})$, with mean $\theta^{*}$ and standard deviation equal to $\sigma_{p}=$ $0.25\times \theta^{*}$.

Bivariate distributions of parameter pairs were estimated using kernel density estimation. Highest density regions (HDRs) for a given probability level were computed and visualized in the corresponding 2D space. The HDR are the smallest regions $R_{\alpha }$ of ${\mathbb{R}}^{2}$ such that if $C_{\alpha }(c)$ represents a contour of constant probability density p(θ)=c enclosing a probability of 1−α then:

$$C_{\alpha }(c)=\{\theta :\theta \in {\mathbb{R}}^{2},p(\theta )=c\}$$ (12)

$$R_{\alpha }(c)=\{\theta :\theta \in {\mathbb{R}}^{2},p(\theta )\geq c\}$$ (13)

$$\int_{R_{\alpha }(c)}p(\theta )d\theta =1-\alpha$$ (14)

The region $R_{\alpha }(c)$ could be, of course, a disconnected region composed of more sub-regions $R_{\alpha_{i}}(c)$ such that:

$$R_{\alpha }(c)=\{\theta \in {\mathbb{R}}^{2}:\int_{{⋃}_{i}R_{\alpha_{i}}(c)}p(\theta )d\theta =1-\alpha \}$$ (15)

### Parameter sampling from the initial distributions π(θ)

For each simulation *i* of the ABC-SMC algorithm and at *p* = 0, the seven free model parameters were sampled from a uniform probability distribution on a pre-specified interval using the Latin Hypercube Sampling (LHS).

LHS is a stratified sampling method ensuring good coverage of the input space, especially useful in high-dimensional problems where random sampling might miss important regions. It works by dividing the range of each input parameter into *n* equal intervals (creating a grid or hypercube), constructing a Latin square of size n x n in the bidimensional case, and then randomly sampling a point within each grid square of the Latin square. A uniform probability distribution was used for each parameter on a pre-specified interval.

The LHS sampling was implemented using the “LatinHypercubeSampling” package [25] in Julia.

Random numbers *u* between 0 and 1 were generated using the Latin Hypercube Sampling procedure, and for each *S*-vector u~U(0,1), the s-th element of the generated parameter $\theta^{*}$ was obtained as follows:

$$\theta_{s}^{*}=m_{s}+u_{s}\times [M_{s}-m_{s}],s=1,\ldots ,S$$ (16)

where *m*_*k*_ and *M*_*k*_ are, respectively, the minimum and maximum values of the domain of the s-th element (see Table 2 for the adopted values).

**Table 2:** Minimum and Maximum values of the domains of the free model parameters.

Parameter	Minimum	Maximum
*k*_*leu*1_ [/min]	0.001	0.2
*γ* [#]	100	10^7^
*λ* [#]	0	6
*k*_*TL*_ [min]	10	10^4^
*k*_*dis*_ [#]	0.5	1
*k*_*leu*2_ [/min]	0.0001	0.1
*t*_*dly*_ [min]	60	1440

## Results

### Parameter estimation for the Experiment CC (FPR1 genotype CC)

**Fig 2:**
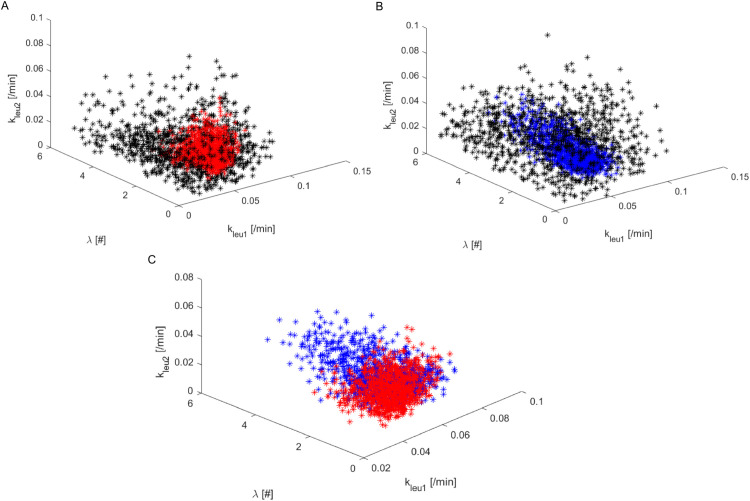
Scatter plot of the accepted parameters from the first and last population. Panel A: 3D scatter plot of the accepted free parameters $\left\{k_{leu1},k_{leu2},\lambda \right\}$ for “Experiment CC” with in black the parameter triplets obtained from the first population $\epsilon_{1}$ and in red the triplets obtained from the last step of the ABC-SMC algorithm, according to the “Bins” criterion. Panel B: 3D scatter plot of the accepted free parameters $\left\{k_{leu1},k_{leu2},\lambda \right\}$ for “Experiment CC” with in black the parameter triplets obtained from the first population $\epsilon_{1}$ and in blue the triplets obtained from the last step of the ABC-SMC algorithm, according to the “Quadrants” criterion. Panel C: 3D scatter plot of the triplets $\left\{k_{leu1},k_{leu2},\lambda \right\}$ from the last populations according to both criteria.

Fig 2, panels A (“Bins” criterion) and B (“Quadrants” criterion), shows the scatter plots of the accepted triplets ($k_{leu1},k_{leu2}$, *λ*) from “Experiment CC”, distinguishing between the set from the population $\epsilon_{1}$ (black points) and from the last population (red points for the “Bins” criterion, blue points for the “Quadrants” criterion). Panel C shows the accepted theta from the final population for the two criteria.

**Fig 3:**
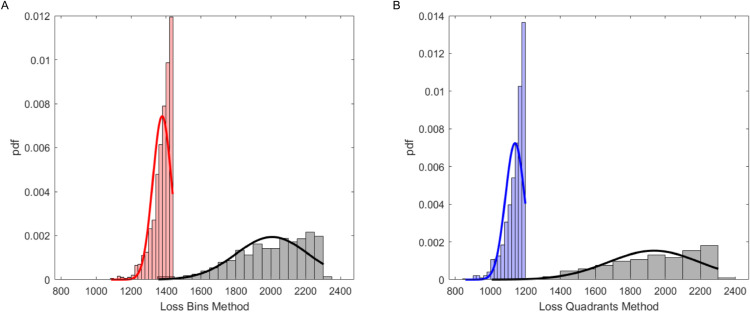
Loss distributions. Panel A: Distribution of the $Loss_{B_{i}}(\theta )$ from the *N*_*SMC*_ accepted θs with the “Bins” method. Panel B: Distribution of the $Loss_{Q_{i}}(\theta )$ from the *N*_*SMC*_ accepted θs with the “Quadrants” method.

It is evident that the final θs from the two criteria lie in the same region, even if parameter *λ* assumes larger values in correspondence of the “Quadrants” method (Panel B). In Panel C of Fig 2 the accepted final points according to the two criteria appear to be superimposed in the 3D space. From the scatter plot it appears that the point cloud identified with the “Bins” method lies within the point cloud identified with the “Quadrants” method. Fig 3, panels A and B, shows the empirical probability distribution of the two losses derived from the “Bins” and “Quadrants” method, respectively, in correspondence of the first ($\epsilon_{1}$, grey bars) and last step (red for “Bins” and blue for “Quadrants” bars) of the ABC-SMC algorithm.

The “Quadrants” method provides lower losses than the “Bins” method, with a higher acceptance rate. The final step for the “Bins” method was in correspondence of P=4, whereas five populations were considered for the “Quadrants” method. The particle number *N*_*SMC*_ was set to 1000. The entire procedure took 2 days for the “Bins” method, resulting in a total of 15340 simulations with $\epsilon_{1}$ (acceptance rate equal to 6.5%), 3220 simulations with $\epsilon_{2}$ (acceptance rate equal to 31%), 9360 simulations with $\epsilon_{3}$ (acceptance rate equal to 10.7%) and 110080 with $\epsilon_{4}$ (acceptance rate equal to 0.9%).

The “Quadrants” method required two days and 6140 simulations with $\epsilon_{1}$ (acceptance rate: 16.3%), 2100 simulations with $\epsilon_{2}$ (acceptance rate: 47.6%), 2860 simulations with $\epsilon_{3}$ (acceptance rate: 35%), 8040 simulations with $\epsilon_{4}$ (acceptance rate: 12.4%) and 65320 with $\epsilon_{5}$ (acceptance rate: 1.5%). The “Quadrants” method proved to be more efficient due to its coarser nature.

**Fig 4:**
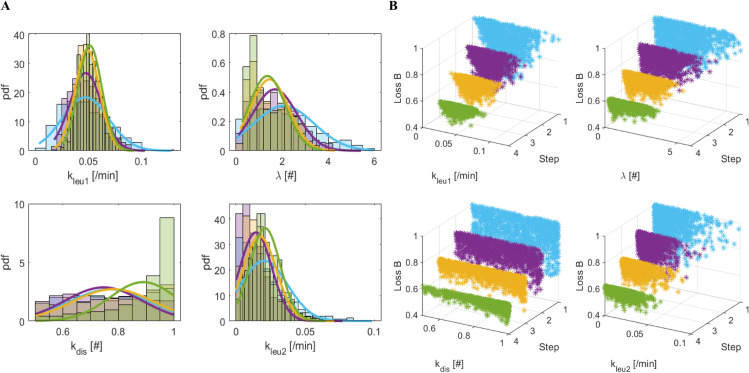
“Bins” marginal distributions from $\pi (\theta ,d(x_{0},x^{*})<\epsilon_{k})$. Panel A. Marginal distributions of $\pi (\theta ,d(x_{0},x^{*})<\epsilon_{k})$ (k=1,.,4) for parameters *k*_*leu*1_, *λ*, *k*_*dis*_ and *k*_*leu*2_, derived from the ABC-SMC algorithm for the “Bins” method. Panel B. Normalized losses (relative to their maximum value) obtained in correspondence of the 1000 accepted θs in the four populations dependent on $\epsilon_{k}$, k=1,.,4. In both panels: light blue for *k* = 1; purple for *k* = 2; yellow for *k* = 3; green for *k* = 4.

**Fig 5:**
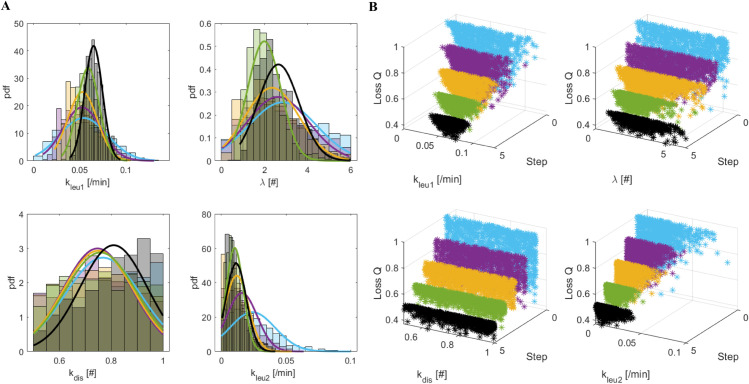
“Quadrants” marginal distributions from $\pi (\theta ,d(x_{0},x^{*})<\epsilon_{k})$. Panel A. Marginal distributions of $\pi (\theta ,d(x_{0},x^{*})<\epsilon_{k})$ (k=1,.,5) for parameters *k*_*leu*1_, *λ*, *k*_*dis*_ and *k*_*leu*2_, derived from the ABC-SMC algorithm for the “Quadrants” method. Panel B. Normalized losses (relative to their maximum value) obtained in correspondence of the 1000 accepted θs in the five populations dependent on $\epsilon_{k}$, k=1,.,5. In both panels: light blue for *k* = 1; purple for *k* = 2; yellow for *k* = 3; green for *k* = 4; black for *k* = 5.

Figs 4 and 5 illustrate, for the most sensitive model parameters (*k*_*leu*1_, *λ*, *k*_*dis*_, *k*_*leu*2_) the intermediate and final distributions (Panels A) obtained when the “Bins” and “Quadrants” methods are applied, respectively. The distributions tend to converge towards regions of higher probability, becoming increasingly concentrated. The remaining parameters exhibit relative non-informative distributions, indicating their low sensitivity within the model: the loss values (Panels B) decrease passing from the initial (ski-blue points) to the final distribution (green for the “Bins” method and black for the “Quadrants” method).

Tables 3 and 4 report the mean and standard deviation of the parameters of the intermediate and final distributions, $\pi (\theta ,d(x_{0},x^{*})<\epsilon_{k})$, with k=1,.,4 for the “Bins” methods and with k=1,.,5 for the “Quadrants” method, respectively. The mean values of the final marginal distributions obtained with the two methods are comparable, despite the large initial ranges considered for each parameter (Table 2). Fig 2 in S1 Text shows the posterior distribution of the difference $D_{k}=Q_{k}--B_{k}$ for the generic parameter *k*. This distribution was constructed by calculating the difference between the corresponding ABC-SMC samples from the two final posterior distributions, *Q*_*k*_ (from the “Quadrants” method) and *B*_*k*_ (from the “Bins” method), i.e., $D_{k}^{\left(s\right)}=Q_{k}^{\left(s\right)}--B_{k}^{\left(s\right)}$ for each sample *s*. The 95% Credible Intervals (CIs) of the resulting *D*_*k*_ posterior distributions have also been displayed. Since all of these credible intervals contain the value zero (the null hypothesis of no difference), and this finding holds even when considering the narrower 90% credible intervals, the posterior evidence does not support the conclusion that the two methods yield credibly different estimates for each one of the *k* parameters.

**Table 3:** Results from the ABC-SMC algorithm for the “Bins” method. Mean and Standard deviation (SD) of the parameter intermediate distributions, $\pi (\theta ,d(x_{0},x^{*})<\epsilon_{k})$, k=1,.,4.

	$\epsilon_{1}$		$\epsilon_{2}$		$\epsilon_{3}$		$\epsilon_{4}$	
	Mean	SD	Mean	SD	Mean	SD	Mean	SD
*k*_*leu*1_ [/min]	0.047	0.022	0.047	0.015	0.049	0.011	0.051	0.011
*γ* [#]	4.598$\times 10^{6}$	2.879$\times 10^{6}$	4.317$\times 10^{6}$	2.758$\times 10^{6}$	4.480$\times 10^{6}$	2.892$\times 10^{6}$	4.302$\times 10^{6}$	2.817$\times 10^{6}$
*λ* [#]	2.077	1.305	1.682	0.954	1.453	0.821	1.356	0.783
*k*_*TL*_ [min]	4992.868	2900.151	4993.812	2705.439	5527.598	2633.165	5113.338	2709.754
*k*_*dis*_ [#]	0.770	0.148	0.746	0.139	0.781	0.147	0.889	0.120
*k*_*leu*2_ [/min]	0.020	0.017	0.014	0.011	0.017	0.012	0.021	0.011
*t*_*dly*_ [min]	854.852	383.157	785.498	383.021	875.234	363.931	812.236	357.513

**Table 4:** Results from the ABC-SMC algorithm for the “Quadrants” method. Mean and standard deviation (SD) of the parameter intermediate distributions, $\pi (\theta ,d(x_{0},x^{*})<\epsilon_{k})$, k=1,.,5.

	$\epsilon_{1}$		$\epsilon_{2}$		$\epsilon_{3}$		$\epsilon_{4}$		$\epsilon_{5}$	
	Mean	SD	Mean	SD	Mean	SD	Mean	SD	Mean	SD
*k*_*leu*1_ [/min]	0.054	0.026	0.052	0.021	0.053	0.016	0.059	0.012	0.065	0.010
*γ* [#]	4.831$\times 10^{6}$	2.949$\times 10^{6}$	4.482$\times 10^{6}$	2.778$\times 10^{6}$	4.168$\times 10^{6}$	2.921$\times 10^{6}$	4$\times 10^{6}$	3.025$\times 10^{6}$	1.565$\times 10^{6}$	2.643$\times 10^{6}$
*λ* [#]	2.847	1.564	2.628	1.432	2.356	1.253	1.996	0.765	2.650	0.949
*k*_*TL*_ [min]	5189.134	2850.595	4860.543	2703.656	5263.356	2771.888	5597.851	2576.255	5857.400	2429.315
*k*_*dis*_ [#]	0.768	0.146	0.747	0.133	0.750	0.136	0.758	0.139	0.809	0.129
*k*_*leu*2_ [/min]	0.024	0.017	0.016	0.012	0.012	0.009	0.010	0.007	0.012	0.008
*t*_*dly*_ [min]	829.425	403.817	753.245	384.876	798.594	391.084	778.698	385.142	719.784	420.020

**Fig 6:**
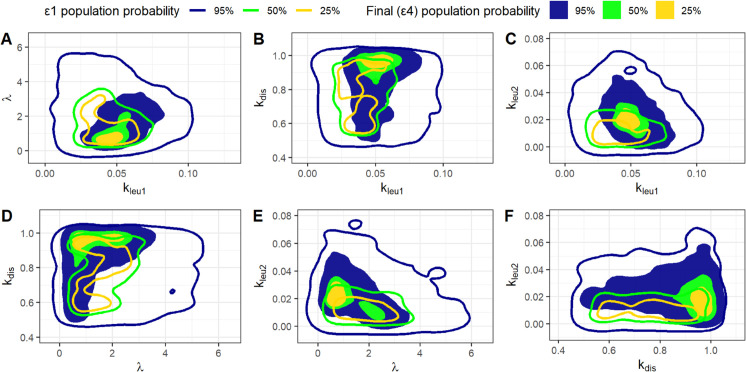
HDR from the “Bins” method. High density regions from bidimensional distributions obtained with the “Bins” method for each pair of the (*k*_*leu*1_, *λ*, *k*_*dis*_, *k*_*leu*2_) parameter vector. Continuous lines represents regions from the distributions obtained with the initial tolerance $\epsilon_{1}$ whereas filled regions are derived from the target final distributions.

**Fig 7:**
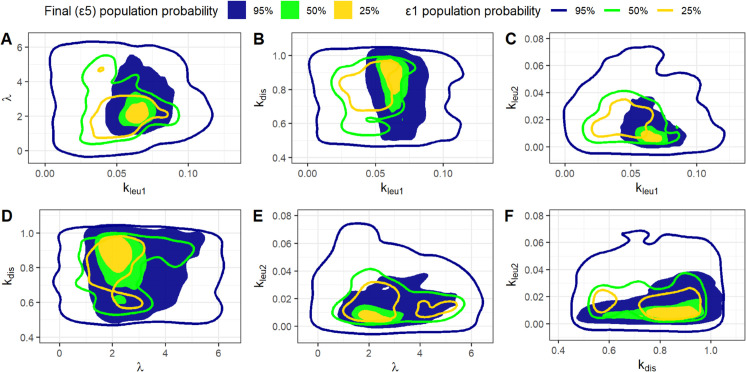
HDR from the “Quadrants” method. High density regions from bidimensional distributions obtained with the “Quadrants” method for each pair of the (*k*_*leu*1_, *λ*, *k*_*dis*_, *k*_*leu*2_) parameter vector. Continuous lines represents regions from the distributions obtained with the initial tolerance $\epsilon_{1}$ whereas filled regions are derived from the target final distributions.

Figs 6 and 7 show the (1-*α*)% High Density Regions (HDRs) for the most sensitive free model parameters (*k*_*leu*1_, *λ*, *k*_*dis*_, *k*_*leu*2_), for the “Bins” and “Quadrants” methods, respectively. HDRs are shown for 25% (yellow), 50% (green), and 95% (blue) confidence levels. Continuous lines represent HDRs calculated with the initial tolerance $\epsilon_{1}$, while filled regions show the tighter HDRs from the final, target distributions. The two methods yield similar regions.

The two figures illustrate that the regions generated from the target distribution are entirely encompassed by those derived from the initial distribution (corresponding to $\epsilon_{1}$). This outcome is not inherently guaranteed, particularly if the parameter space is not comprehensively explored. An incomplete exploration could inadvertently exclude regions that might contain model realizations very close to the observed data. Should such initially overlooked parameters be accepted in subsequent steps of the algorithm, the final regions would not be completely contained within the initial ones. It appears that employing the Latin Hypercube Sampling procedure for sampling from the a priori distribution helps to prevent leaving such regions unexplored.

Figs 3 and 4 in S1 Text show the scatter plot of each pair of parameters estimates from the last population with the estimated linear regression model and the relative Pearson correlation coefficients, *r*. Tables 2 and 3 in S1 Text reports the P values of the *r* coefficients.

**Fig 8:**
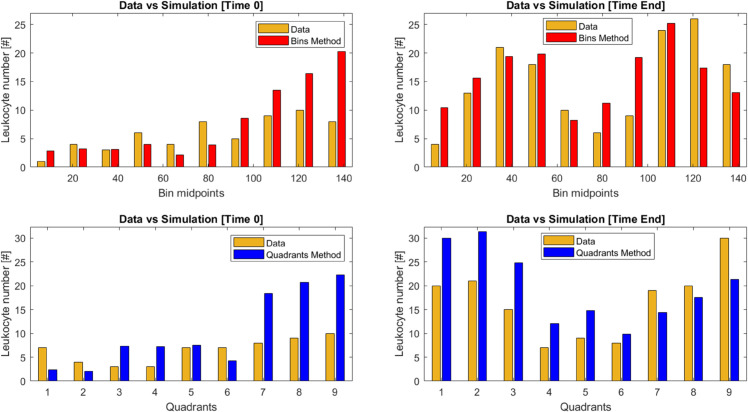
Statistic comparisons. Panel A: observed (gold) and predicted (red) number of leukocytes along the horizontal domain for the “Bins” criterion in correspondence with frame 0 (24 h from the beginning of the experiment). Panel B: observed (gold) and predicted (red) number of leukocytes along the horizontal domain for the “Bins” criterion in correspondence with frame 720 (48 h from the beginning of the experiment). Panels C and D: numbers of leukocytes (gold for observed and blue for predicted) in the nine portions of the considered domain for the “Quadrants” method for the frames at 24 h and 48 h, respectively. The red and blue bars represent the averages of the absolute leukocyte frequencies calculated from the realizations obtained with the accepted parameter sets of the final target population.

Fig 8 shows the observed (gold bars) and estimated (red and blue bars) frequencies of leukocytes obtained for the “Bins” (panels A and B) and “Quadrants” (panels C and D) method. Panels A and C correspond to the first frame (24 hours after the start of the experiment) while panels B and D correspond to the last frame of the movie (at 48 hours from the beginning of the experiment). The red and blue bars in figure represent the average absolute leukocyte frequencies calculated for each bin (red) and quadrant (blue), respectively. These averages were derived from the realizations obtained with the accepted parameter sets of the final target population. The figure demonstrates a good qualitative agreement with the experimental data, compatible with the smallest considered threshold $\epsilon_{T}$, with the “Bins” method exhibiting a slightly better fit to the observations.

Both the criteria tend to overestimate the number of leukocytes in the rightmost compartment of the domain at the beginning of the experiment (frame 0). At frame 720 (48 h from the beginning of the experiment), while the “Bins” method estimates adapt well to the observed frequencies along the entire horizontal axis, the “Quadrants” method fails to accurately reproduce the observed frequencies in the leftmost quadrants of the domain. For both methods, the Chi-squared test was not significant, indicating no evidence to reject the null hypothesis of similarity between each pair of distributions. For the “Bins” method, Chi-squared values were 9.2 (P = 0.41) for frame 0 and 10.81 (P = 0.29) for frame 720. For the Quadrant method, Chi-squared values were 14.4 (P = 0.07) and 10.03 (P = 0.26) for frame 0 and frame 720, respectively. The Root Mean Squared Error (RMSE) values for the “Bins” method were 4.8 and 5.4 for frame 0 and frame 720, respectively. The corresponding values for the Quadrant method were 7.13 and 7.30, indicating poorer predictive performance compared to the “Bins” method.

**Fig 9:**
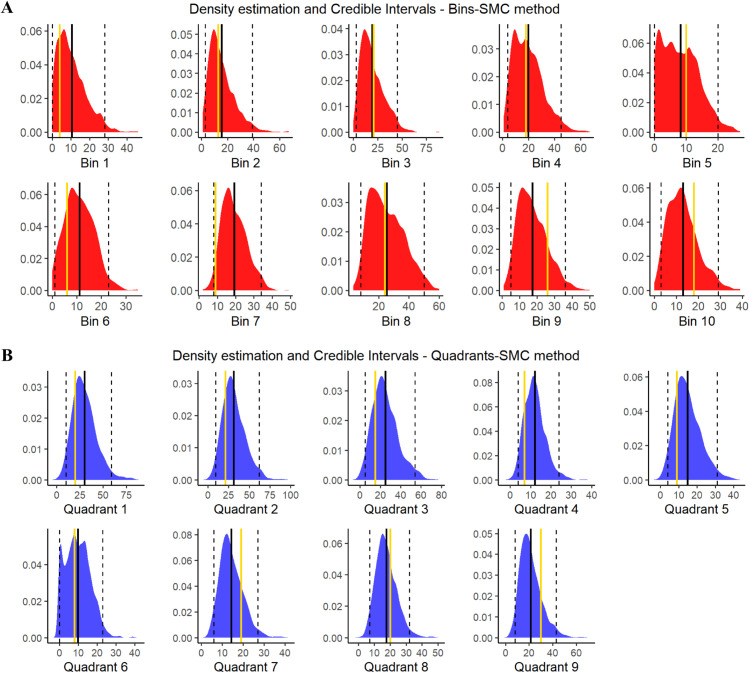
Statistic distributions. Distributions of the estimated leukocyte frequencies in correspondence of each bin (red curves) and of each quadrant (blue curves) obtained with the θs of the final distributions. Gold lines represents the observed values; black continuous lines are the averages of the distributions; dotted black lines are the upper and bound limits of 95% credible regions.

Fig 9 shows the variability around the estimates of the leukocyte frequencies obtained with the two methods (“Bins” and “Quadrants”). The Figure reports the observed values (gold lines) and the kernel density estimations of the frequency distributions of the estimated values obtained from the final θs are reported in correspondence of each bin (panel A) and each quadrant (panels B). Black lines represent the averages of the distributions, and black dotted lines delimit the 95% credible regions.

### Comparison between “Experiment CC” (FPR1 genotype CC) and “Experiment CA” (FPR1 genotype CA)

The key parameter for comparing the two experimental situations is *λ*, as defined in Eq 4 of [16]. This parameter reflects the amplification of leukocyte migration in response to higher annexin concentrations. It can distinguish between the behaviors of two different leukocyte populations based on their sensitivity to annexin, particularly those with the FPR1 genotype CC versus CA.

Table 5 reports the average values of the marginal posterior distributions of parameter θ obtained from the two experiments. Values for the CC Genotype are from Tables 3 and 4. Fig 5 in S1 Text shows the delta distributions obtained for “Experiment CA”, mirroring results for “Experiment CC”, shown in Fig 2 in S1 Text, except for parameters *λ* and *k*_*leu*2_ which result to be different between the two methods, “Bins” and “Quadrants”, with 95% Credibility Intervals containing the zero value. Same results are obtained when the 90% CIs are considered.

**Table 5:** Average values (standard deviation) of the final θ’s for the “Bins” and “Quadrants” methods in the two experiments.

CC Genotype			CA Genotype	
Parameter	“Bins”	“Quadrants”	“Bins”	“Quadrants”
*k*_*leu*1_	0.051 (0.011)	0.065 (0.010)	0.114 (0.008)	0.120 (0.008)
*γ*	4.302$\times 10^{6}$ (2.817$\times 10^{6}$)	1.565$\times 10^{6}$ (2.643$\times 10^{6}$)	5.474$\times 10^{6}$ (2.561$\times 10^{6}$)	4.221$\times 10^{6}$ (2.756$\times 10^{6}$)
*λ*	1.356 (0.783)	2.650 (0.949)	0.304 (0.045)	0.598 (0.409)
*k*_*TL*_	5113.338 (2709.754)	5857.4 (2429.315)	4933.728 (2813.906)	5024.414 (2741.284)
*k*_*dis*_	0.889 (0.120)	0.809 (0.129)	0.726 (0.136)	0.736 (0.132)
*k*_*leu*2_	0.021 (0.011)	0.012 (0.008)	0.0156 (0.004)	0.005 (0.003)
*t*_*dly*_	812.236 (357.513)	719.784 (420.020)	620.976 (351.326)	796.441 (378.572)

When comparing the two genotypes within the “Bins” Method, all the 95% Credible Intervals (CIs) for the delta distributions (the posterior distribution of the difference between genotypes) include zero, except for parameters *k*_*leu*1_ and *λ*. Parameter *k*_*leu*1_, which represents the leukocyte accrual rate in the right chamber, is credibly larger in “Experiment CA” compared to “Experiment CC” (0.114±0.008 and 0.051±0.011, respectively). This result is notable despite fewer leukocytes subsequently being found in the left chamber. The magnitude of parameter *λ* distinguishes between isotropic and anisotropic motion. The posterior mean ± standard deviation of *λ* for “Experiment CC” is 1.356±0.783, which is credibly higher than the posterior mean of 0.304±0.045 obtained for “Experiment CA". The finding that these differences persist when considering the 90% CIs confirms the strength of the evidence for a credible difference between the experiments for these two parameters. Conversely, the 90% CIs still include zero for the remaining parameters, reinforcing the conclusion of no credible difference. Fig 6 in S1 Text shows the distributions of the differences between each pair of parameter distributions along with the respective 95% Credible Intervals. Similar results regarding differences between genotypes are obtained within the “Quadrants” method: Parameter *k*_*leu*1_ is credibly larger in “Experiment CA” than in “Experiment CC” (0.120±0.008 and 0.065±0.010, respectively), and parameter *λ* is credibly smaller in the “Experiment CA” genotype than in genotype “Experiment CC” (0.598±0.409 and 2.650±0.949, respectively). The distributions of the parameter differences between the two experiments for the “Quadrants” method are shown in Fig 7 in S1 Text.

**Fig 10:**
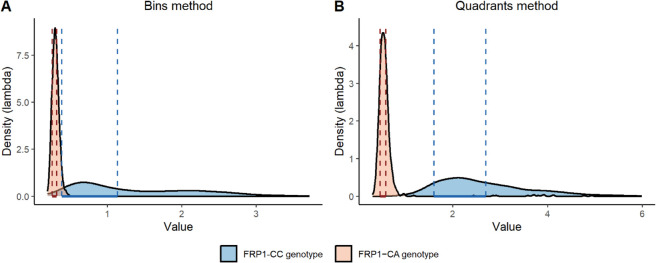
Parameter λ distributions by experiment. Kernel density estimates of the empirical posterior distributions for parameter *λ* in the “Bins” method (Panel A) and in the “Quadrants” method (Panel B). Light blue areas are related to “Experiment CC” whereas light brown areas are related to “Experiment CA”. The regions delimited by dashed lines represent 50% HDI.

Fig 10 shows the smoothed shapes of the empirical marginal posterior distributions of the parameter *λ*, along with the corresponding High Density Intervals (HDI) (dashed lines) containing 50% of the mass of the distributions, both for the “Bins” (Panel A) and “Quadrants” (Panels B) method in the two experimental situations. The figure clearly shows that the parameter *λ* effectively distinguishes between the two experiments, resulting in a distribution more concentrated on smaller values in “Experiment CA”. Lower values of *λ* indicate reduced sensitivity to the chemoattractant, leading to a decreased likelihood of anisotropic leukocyte movement. Table 6 reports the 50%, 75% and 95% credible intervals for parameter *λ* for both methods.

**Table 6:** High density intervals (or Credible Intervals) containing the 50%, 75% and 95% of the mass of the posterior distributions approximated by Kernel Density estimates.

Parameter	HDI - “Bins”		HDI - “Quadrants”	
λ	CC genotype	CA genotype	CC genotype	CA genotype
50%	0.388-1.138	0.262-0.320	1.606-2.701	0.474-0.590
75%	0.381-2.043	0.246-0.346	1.382-3.314	0.444-0.642
95%	0.376-2.848	0.218-0.389	1.245-4.723	0.374-0.751

Tables 7 and 8 present the 90% HDIs for all free model parameters. HDIs were derived from the posterior distributions corresponding to both the “Bins” and “Quadrants” method. Each table also includes the percentage of overlap within each interval. The degree of overlap between these credible intervals serves as an indicator of similarity or difference between the parameters across the two experiments. A quantification of overlapping is assessed by computing the ratio of the overlap range to the total width of the HDIs. Also this analysis reveals that, with the exception of parameters *k*_*leu*1_ and *λ* all other parameters exhibit substantial overlap for both the “Bins” and “Quadrants” methods. This significant overlap suggests that these parameters are not practically different when estimated using the two experimental situations.

**Table 7:** 90% High density intervals (HDIs) of the model parameter posterior distributions from the “Bins” method, along with the percentage of overlap relative to the HDI.

Parameter	90% HDI		% Overlap	
	CC genotype	CA genotype	CC genotype	CA genotype
*k*_*leu*1_	0.033-0.068	0.101-0.127	0%	0%
*γ*	230666-8315419	1554457-9708205	83.6%	82.9%
*λ*	0.381-2.576	0.232-0.374	0%	0%
*k*_*TL*_	285.2-8700.5	217.1-8852.7	100%	97.4%
*k*_*dis*_	0.688-1	0.501-0.924	75.8%	55.8%
*k*_*leu*2_	0.003-0.037	0.008-0.022	41.1%	100%
*t*_*dly*_	255.7-1381.4	70.0-1126.5	77.3%	82.4%

**Table 8:** 90% High density intervals (HDIs) of the model parameter posterior distributions from the “Quadrants” method, along with the percentage of overlap relative to the HDI.

Parameter	90% HDI		% Overlap	
	CC genotype	CA genotype	CC genotype	CA genotype
*k*_*leu*1_	0.048-0.080	0.107-0.134	0%	0%
*γ*	5784-6473663	645.5-8292720	100%	78%
*λ*	1.34-4.20	0.40-0.701	0%	0%
*k*_*TL*_	2515.2-9930.2	1367.3-9963.7	100%	86.3%
*k*_*dis*_	0.607-1	0.529-0.948	86.8%	81.4%
*k*_*leu*2_	0.001-0.0229	0.001-0.01	37.9%	94.7%
*t*_*dly*_	185-1436.9	220.8-1412.1	95.2%	100%

For both methods, the new leukocyte accrual rate in the right chamber (*k*_*leu*1_) is approximately twice as high in the CA genotype compared to the CC genotype. This observation aligns with the higher number of leukocytes found in the right chamber during the CA experiment. Their migration is primarily isotropic, preventing them from effectively moving towards tumor cells located in the microchip’s left compartment. Parameter *λ*, which expresses the tendency of leukocytes to migrate towards regions with a higher attractor concentration, is, as expected, significantly higher in “Experiment CC” than in “Experiment CA”. While the mean values of all other parameters generally align with expectations, they don’t seem to differ significantly between the two experimental procedures. Parameter *k*_*leu*2_ is on average greater in the CC genotype experiment. Here, leukocytes appear more frequently in the left chamber, likely driven by their sensitivity to annexin. Parameter *γ*, which represents the annexin detection threshold for leukocytes, does not appear to be sensitive to the experiment type, given its almost completely overlapping HDIs. Parameters *k*_*TL*_, *k*_*dis*_, and *t*_*dly*_ hold very little relevance in the CA genotype experiments. If leukocytes are unable to perceive annexin, the values of these parameters have no impact on leukocyte dynamics. Consequently, their estimated values span their entire range of variation, resulting in average values very close to the interval average.

**Fig 11:**
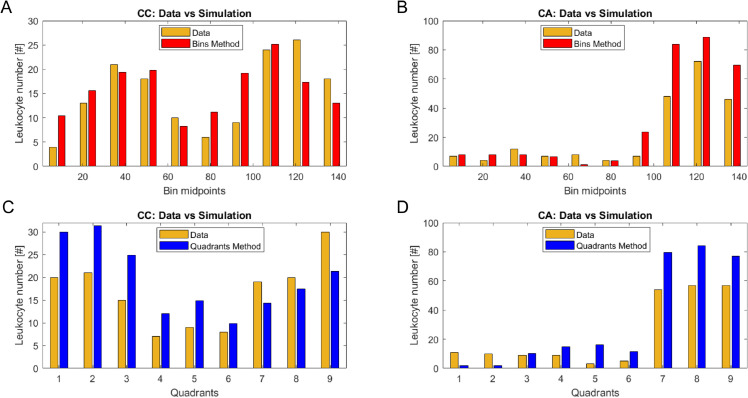
Statistic comparisons by experiment. Panels A and B: observed (gold) and predicted (red) leukocyte number in each of the ten bins (created by dividing the horizontal axis according to the “Bins” method) at 48 h for “Experiment CC” (CC genotype) and “Experiment CA” (CA genotype), respectively. Panels C and D: observed (gold) and predicted (blue) leukocyte number in each of the nine quadrants (created by dividing the domain according to the “Quadrants” method) at 48 h for the same experiments.

Fig 11 shows the fitting results related to frame 720 (48 hours from the beginning of the experiment). Panels with gold and red bars refer to the “Bins” method, with Panel A for “Experiment CC” (CC genotype) and Panel B for “Experiment CA” (CA genotype). Conversely, panels with blue bars are related to the “Quadrants” method, with Panel C reporting results from “Experiment CC” and Panel D from “Experiment CA”.

**Fig 12:**
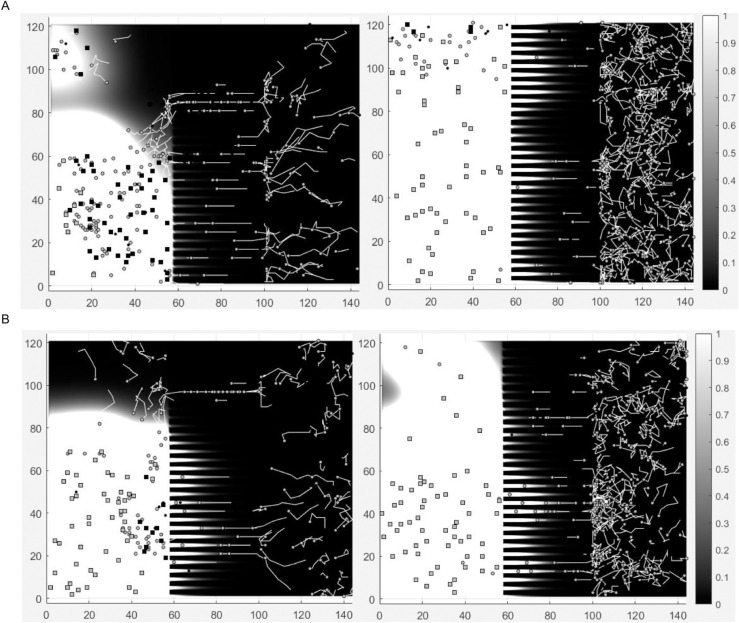
Model simulation by experiment. Panel A: final simulation states obtained with the “Bins” method; panel B: final simulation states obtained with the “Quadrants” method. For both methods, images on the left represent the simulations describing the behavior of the leukocytes (circles) with the FPR1 genotype CC (“Experiment CC”), able to activate an anticancer immune response by recognizing and binding annexin A1 (color gradient). They exhibit an anisotropic (directed, non-random) movement towards the tumor cells (squares) located in the left chamber of the chip. In “Experiment CA” (right panels), the leukocytes with the heterozygous *FPR*1^*CA*^ genotype show reduced movement and interaction and exhibit an isotropic movement within the rightmost chamber of the chip.

Movies 1-4 in S2 File, provided as supplementary information, illustrate representative simulations of Experiments CC (Movies 1 and 3 in S2 File) and CA (Movies 2 and 4 in S2 File) for both the “Bins” method (Movies 1 and 2 in S2 File) and the “Quadrants” method (Movies 3 and 4 in S2 File), using a θ sampled from the final distributions. Fig 12 shows the last frames (at 48 hours from the beginning of the experiment) of the four movies; panel A depicts the final simulation states obtained with the “Bins” method, whereas panel B represents the corresponding states obtained with the “Quadrants” method. Images on the left are relative to experiment CC, where the invasion of the left chamber by the leukocytes (circles) is evident. In this experiment, leukocyte movement is driven by Annexin concentration, represented by the gradient scale. Images on the right refer to experiment CA, where almost all of the leukocytes, which have the FPR1 genotype CA, show an isotropic (random, non-directional) movement inside the rightmost chamber of the chip.

## Discussion

Microfluidic cell Co-Culture, Tissue Co-Culture, Organ-on-Chip and Cancer-on-Chip technologies are valuable tools for precision medicine, drug development and testing, as well as patient screening. These technologies can reproduce complex interactions between different cell types in vitro, mimicking the human tissue microenvironment. By combining mathematical models with data from sensors, microcinematography or images, researchers can formalize and quantitatively interpret experimental observations. However, realistic simulations often require multiscale models [26–32]. Computational models leveraging cellular automata, agent-based modeling, stochasticity, pharmacodynamics, and pharmacokinetics have the potential to plausibly mimic the behavior of cellular subtypes cultured in the microenvironment of the chip and to quantify drug-specific parameters, including tissue distribution, solubility, binding affinity, membrane permeability, and clearance. This approach, involving a fine granularity, naturally incurs a cost in terms of computational requirements and model complexity.

In this work, we extend the hybrid agent-based model presented in [16] to more accurately reflect the experimental setup from which it was derived. This model represents the migration of mononuclear blood cells toward pre-treated cancer cells. It also simulates the interactions between these cell types. The work in [16] aimed to improve upon a previous model [33]. That model consisted of a discrete-in-continuous PDE reaction-diffusion model coupled with an ODE model. It described the chemical and cancer cell dynamics observed only in the left chamber of the experimental setup, which focused solely on that chamber. The mathematical models were inspired by a microfluidic cell co-culture experiment [17,18]. In that experiment, pretreated human breast cancer cells and peripheral blood mononuclear cells were cultured [19]. This original in vitro work aimed to demonstrate the ability of specific blood cells to activate an anticancer immune response through the recognition and binding of annexin A1.

In [16], the authors used a hybrid agent-based approach to describe leukocyte dynamics driven by the annexin gradient across the entire video frame domain. The diffusion and consumption of the chemical attractant were described by a discretized reaction-diffusion partial differential equation. Conversely, leukocyte movement over the chip was described using non-isotropic Brownian motion, influenced by the chemical signal gradient established in the environment. Subsequently, in [21], the model was extended to incorporate changes that influence the dynamics of leukocyte/tumor interaction. These changes included a limited lifetime for both leukocytes and cancer cells, which affects the extent of their interaction and gives rise to a bidirectional influence between leukocytes and annexin. A more accurate representation of the chip geometry was also introduced, along with a much more efficient parallelized implementation enabling a large number of simulations in a reasonable execution time.

The scope of this work is to validate a modified version of the model presented in [16,21] using observed data derived from tracking individual leukocytes by microphotographs captured every 2 minutes (for a total of 720 frames) from a fluorescence videomicroscopy of the experiment conducted on the microfluidic chip described above. The quantified output from cell-tracking was used to estimate some key model parameters by means of Approximate Bayesian Computation. Parameter estimation is the core of the present work: it is indeed, in most cases, a difficult task when dealing with AB models, due to their intrinsic complexity (granularity, presence of uncertainty, many parameters, incompletely observed data, lack of relevant measures). Usually, at least for some parameters, plausible ranges of values are known from the literature or stem from biological considerations. In the present work some model parameters were set on the basis of the results from [21]. However, several parameters remain to be estimated or calibrated. Calibration is often very difficult, since parameters may not be independent and calibration would require testing a large number of combinations, while stochasticity must still be taken into consideration. Therefore, efficient methods for sampling or optimization must be considered, as well as metrics that allow to quantify how close the model output is to observed data. In fact, the classical method of Maximum Likelihood Estimation, while desirable for its asymptotic properties, cannot be applied to such a complex stochastic model because the likelihood functions are difficult to write or are analytically intractable. Approximate Bayesian Computation methods have been widely used in recent years because they not only are able to provide a point estimate of the parameter vector but also allow for a quantification of uncertainty and correlation between parameters. In fact, the final scope of ABC approaches is to estimate the posterior distribution of model parameters figuring out what values the model’s parameters are most likely to be, given some observed data. In this work we implemented the ABC method based on the Sequential Monte Carlo approach (ABC-SMC), as described in [23].

This algorithm proved not to be very computationally expensive, requiring 2 days regardless of the method used (“Bins” or “Quadrants”) for “Experiment CC”. The time increased for “Experiment CA” to 4 days for the “Bins” method and 3 days for the “Quadrants” method.

The results obtained seem encouraging: the distributions of the statistics computed in correspondence of the θs from the final distributions for the two methods are quite centered on the values of the statistics computed from observed data (see Fig 9) and the 95% High Density Regions include the observed quantities. Fig 8 also shows a very good approximation of the simulated statistics to the observed statistics, especially for the “Bins” method in correspondence of the last frame, where the predicted bars approximate very well the size of the observed bars along all the horizontal axis of the domain. Also the coefficients of variation of the key model parameters are not very large: 21.6% and 15.4% for *k*_*leu*1_, 13.5% and 15.9% for *k*_*dis*_, 52.4% and 66.7% for *k*_*leu*2_ and 57.7% and 35.8% for *λ*, in correspondence of the “Bins” and “Quadrants” method, respectively. For both methods, the divergence between the observed and predicted statistics appears more evident at the beginning of the experiment and narrows in correspondence of the last frame (at 48 hours).

The overestimation at the beginning of the simulation in the right chamber is likely due to the current model formulation, which allows at most one leukocyte to enter the system at each temporal step. The constant value of the accrual rate must be set to approximate the total number of leukocytes, and their distributions inside the domain, throughout the entire simulation. This approach cannot accurately reflect the dynamic distribution of leukocytes across the domain at different time points. A more adaptable system would have included an additional compartment with an initial number of leukocytes or an accrual rate that varies over time. This would better represent leukocytes already present in the main compartment that gradually move toward the central, end-closed channel, attracted by Annexin A1. This more realistic approach would have helped to avoid overestimating the number of leukocytes in the rightmost part of the domain at the beginning of the simulation.

The parameter estimates obtained with the two methods (“Bins” and “Quadrants”) are comparable. All the 95% and 90% Credible Intervals of the posterior distribution for the difference between the paired parameters contain zero when comparing parameter distributions obtained for the “Experiment CC” condition. For the “Experiment CA” condition, the parameters *λ* and *K*_*leu*2_ are found to be different between the two methods; however, their estimated values are consistent with the experimental setting, supporting the robustness of the modelling approach adopted and of the estimation procedure. Parameter *K*_*leu*2_, which represents the leukocyte accrual rate in the left chamber, is indeed estimated to be lower in the “Experiment CA” than in the “Experiment CC” for both methods. When comparing the two genotypes within both the “Bins” and “Quadrants” methods, the only 95% Credible Intervals for the posterior difference that do not include zero are those related to parameters *k*_*leu*1_ and *λ* (see Figs 6 and 7 in S1 Text). The parameter *k*_*leu*1_, which represents the leukocyte accrual rate in the right chamber, is credibly larger in “Experiment CA” compared to “Experiment CC”. This result occurs even though fewer leukocytes are able to reach the left chamber in “Experiment CA”. Parameter *λ*, quantifying the anisotropic movement of leukocytes, is estimated to be credible smaller in the CA compared with the CC experiment (with credibility intervals, High Density Intervals, lying in different regions of the parameter space), thus agreeing with the original experimental hypothesis [19]: blood cells exhibiting the homozygote CC genotype of the formyl peptide receptor 1 are (better) able to recognize annexin A1 and link with it, activating the anticancer immune response. For the “Quadrants” method Credibility intervals for *λ* in the two experiments are completely disjoint while for the “Bins” method they are only slightly superimposed for 95% probability (see Table 6 and Fig 10). The overlap between the two intervals is 0% when considering a 90% Highest Density Interval (Tables 7 and 8). The parameter *λ* thus appears capable of quantifying the degree of sensitivity of the leukocytes to annexin and consequently the degree of induction of an anti-tumor immune response by chemotherapy: the values within the HDIs derived for “Experiment CA”, approaching the zero, are consistent with the experimental setting in which leukocytes, bringing the FPR1 receptor with CA genotype, are insensitive to annexin. The ABC-SMC approach clearly highlights the differential effect of the parameter in the two experimental situations.

However, it is to be noted that the estimation procedure adopted here is only one of the many possible procedures that could be implemented. For example, while in the present work a single value summarizing the distance between observed data and simulated realizations was defined (sum of the mean square deviations between the model output and the observed data), a categorical approach could have been used, according to which a range of plausible values would be defined for each target criteria [34]. Such an approach could prove particularly suitable in presence of high uncertainty. Still, for both the “Bins” and “Quadrants” methods, it might have been necessary to consider smaller tolerances for the difference between the simulated output and observations within each bin or quadrant. While this could have led to a more accurate (closer to the observed data) prediction, it would have increased the computational cost. This limitation is inherent to the present work.

Comparisons could also be made between various methods for exploring parameter spaces. A full factorial design could be employed, but only in the presence of a limited number of parameters since, by systematically exploring the entire parameter space, the number of combinations could be very large and the problem could become computationally intractable. Fractional factorial design could be used for selecting only a subset of the possible parameter combinations from a fully factorial design. Other sampling methods, such as simple random sampling (with replacement) or Latin Hypercube Sampling (adopted in the present work), are used to avoid full factorial designs. These methods require choosing a prior distribution from which to draw the parameter values, and one might choose probability densities other than the uniform distribution used here. For parameters spanning several orders of magnitude (such as *γ*), employing lognormal rather than uniform distributions for the initial distribution would have provided better coverage of values near the lower bounds. While the current uniform sampling approach yielded robust results, future work could benefit from this refinement in the specification of the priors. Furthermore, instead of simulating the process starting from sampled parameter sets, whatever the sampling method, optimization methods could be employed, which differ from the previous methods because the sets of parameters to be evaluated are not created before the start of the simulations, but simulations are run at parameter values that depend on previous results. Starting from a parameter value we could define the loss function as described in the “Approximate Bayesian Computation for model parameter estimation” subsection, Eqs 8 and 10, and then use a gradient, a quasi-Newton or simplex method to find the optimum theta. Also in the framework of the employed ABC method, different methods (as the ABC-MCMC) or different loss functions (target criteria) or alternative methods to retain the “best” parameters could be considered. Moreover, other likelihood-free methods exist and offer different trade-offs. For example, Synthetic Likelihood is a computationally more efficient alternative. Unlike ABC, which is non-parametric, a common form of synthetic likelihood approximates the distribution of summary statistics using a parametric model, typically a Gaussian distribution. This assumption of normality, while leading to computational efficiency, is a critical dependency that may not always hold true. It’s also important to note that some authors discourage the use of certain synthetic likelihood methods for determining credible or confidence intervals due to their heuristic nature [35–39]. Nevertheless, when the distribution assumption is valid, synthetic likelihood methods can offer a more direct and efficient path to inference. Future work could benefit from a direct comparison of different estimation approaches to explore the balance between computational efficiency and the robustness of their underlying assumptions within the context of our specific model.

## Conclusions

The approach followed in this work is only one of several possibilities and it is clear that the estimated final model parameters from the two methods, which were found to be the best parameter estimates, could not be, and indeed are not, capable of perfectly reproducing the observed patterns.

Other computational approaches, such as different choices of prior distributions for the parameters, different criteria for defining the closeness of the simulations to data, or different parameter estimation methods and modeling formalizations, could have led to different results. In this sense, it would be extremely interesting, and subject of future work, to evaluate different procedures that differ both in terms of target criteria (a different loss function) and estimation strategies (including optimization procedures for finding the optimal parameter value or approximated likelihood-based methods), comparing them (even if it would require extensive computations) based on both computational efficiency and the fidelity of reproduction observed patterns.

## Supporting information

S1 Text Supplementary material. (PDF)

S2 File MOVIES.ZIP. This folder contains Movie 1 (Simulation of Experiment CC, “Bins” method), Movie 2 (Simulation of Experiment CC, “Quadrants” method), Movie 3 (Simulation of Experiment CA, “Bins” method) and Movie 4 (Simulation of Experiment CA, “Quadrants” method)
(ZIP)
