# Supplementary material for: Validation of an agent-based model for cell interactions in a microfluidic chip
Source: PLoS One. 2026 Feb 9;21(2):e0341962. doi: 10.1371/journal.pone.0341962 (PMC12885325; doi:10.1371/journal.pone.0341962)
Supplement: S1 Text — (PDF) [file pone.0341962.s001.pdf]

Supplementary Material

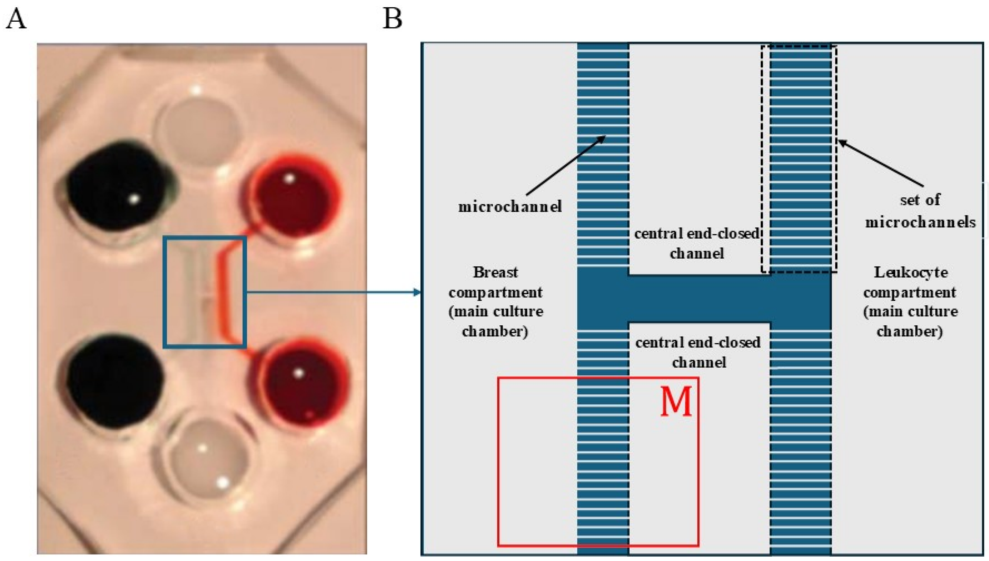

Fig S1. Panel A: real photograph of the microfluidic device as it appears in Businaro et al. [17]. Panel B: 2D-representation of the central part of the chip with the modelled portion (red square, matrix M).

Table S1. Parameters of the Agent Based Model.

Parameter	Description	Units	Value.
Δt	discretization time step	min	4
Δx	discretization along the x axis	μm	12
Δy	discretization along the y axis	μm	12
D	Diffusivity of annexin	$\mu m^2/min$	$1.5 \cdot 10^4$
k_A	annexin production rate	$Mmin^{-1}$	40
k_{XA}	annexin elimination rate	min^{-1}	$5 \cdot 10^{-3}$
L_x	horizontal size of the domain	μm	1707
L_y	vertical size of the domain	μm	1452
r_l	radius of a leukocyte	μm	4
r_c	radius of a tumor cell	μm	10
N_c	number of tumor cells	μm	60
L_c	lifetime of a tumor cell	min	$1000 \cdot 60$
L_L	maximum leukocyte lifetime	min	$144 \cdot 60$
k_{leu1}	normalized rate of new leukocyte accrual in the right chamber	min^{-1}	free
γ	threshold value for migration	#	free
λ	tendency of a leukocyte to migrate towards higher annexin concentrations	#	free
k_{TL}	tumor cells life reducing by leukocytes	min	free
k_{dis}	portion of tumor cells distributed in the lower half of the left chamber	min	free
k_{leu2}	normalized rate of new leukocyte accrual in the left chamber	min^{-1}	free
t_{dly}	mean delay time for the appearance of a leukocyte in the left chamber	min	free

Parameter Estimates Comparison Between “Bins” and “Quadrants” methods for “Experiment CC”

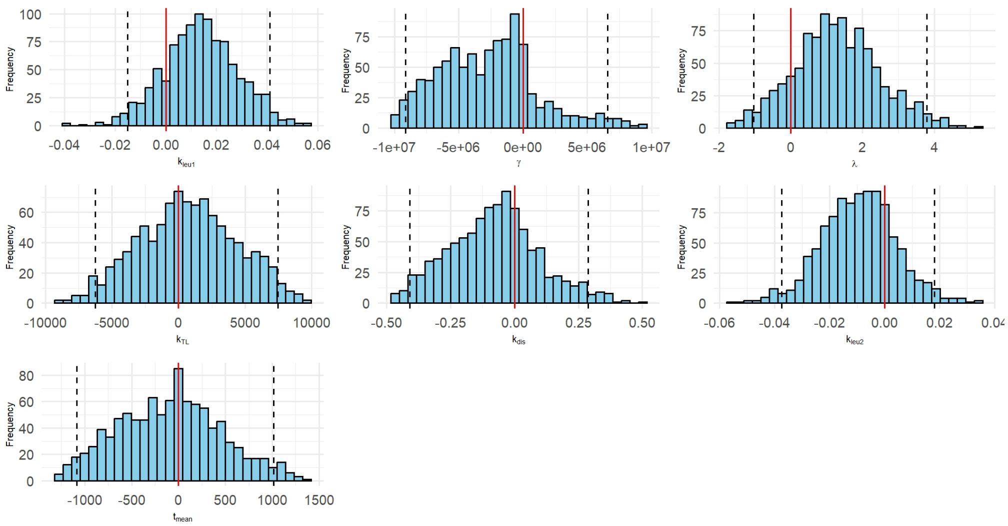

Fig S2. Posterior distributions of the differences $D_k = Q_k – B_k$. Each panel displays the posterior distribution of the difference (D_k) between parameter estimates obtained using the “Bins” (B_k) and “Quadrants” (Q_k) methods for a generic parameter k in “Experiment CC”. The dashed black lines indicate the limits of the 95% Credible Intervals, and the continuous red line marks the zero value. The fact that all Credible Intervals encompass zero suggests that the two methods do not yield significantly different parameter estimates.

Parameter correlations

Figures S3 and S4 show the scatter plot of each pair of parameters estimates from the last population with the estimated linear regression model and the relative Pearson correlation coefficients, r. Tables S2 and S3 reports the P values of the r coefficients.

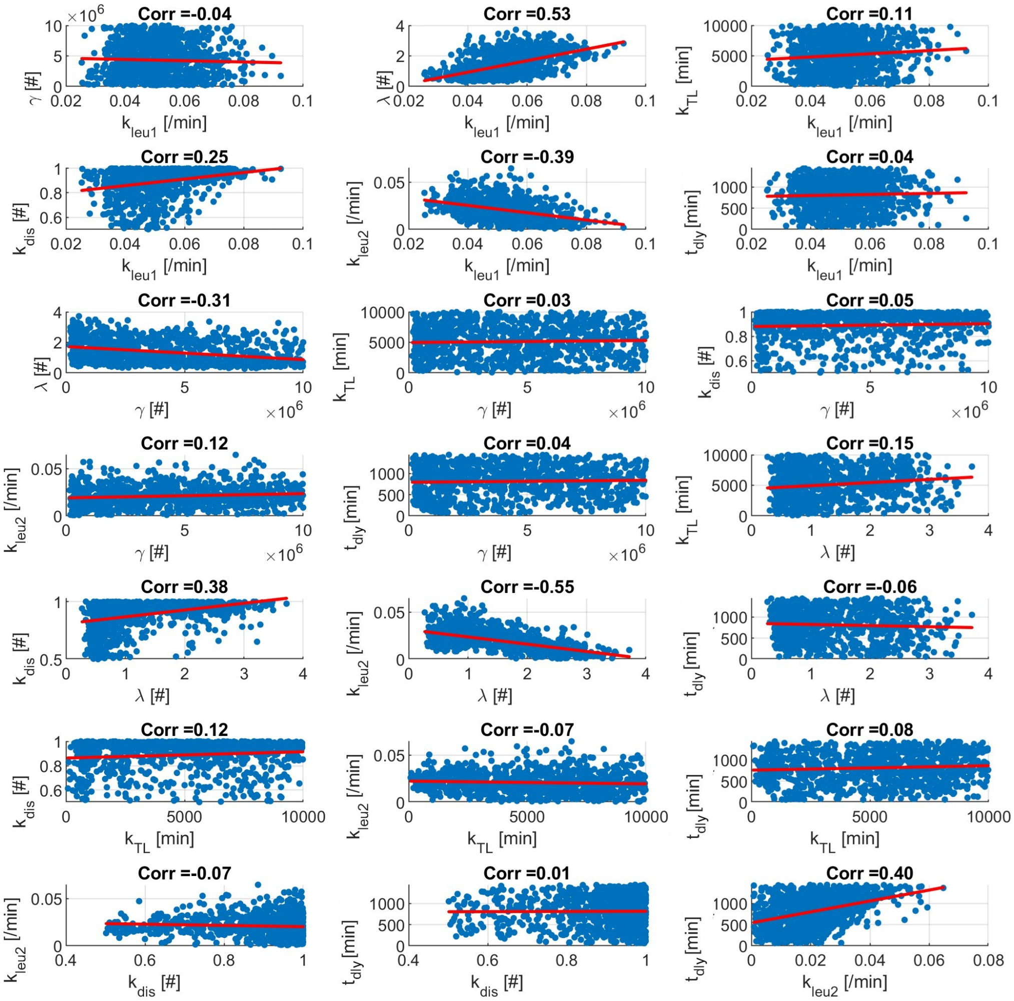

Fig S3. "Bins" method - Scatter plot of each pair of parameter estimates along with the the estimated linear regression curve (red lines). For each plot the Pearson correlation coefficient r is reported on the top.

Table S2. "Bins" method - P-values of the Pearson correlation coefficients.

	k_{leu1}	γ	λ	k_{TL}	k_{dis}	k_{leu2}	t_{dly}
k_{leu1}	1						
γ	0.203	1					
λ	< 0.0001	< 0.0001	1				
k_{TL}	0.001	0.280	< 0.0001	1			
k_{dis}	< 0.0001	0.091	< 0.0001	0.0003	1		
k_{leu2}	< 0.0001	0.0003	< 0.0001	0.023	0.024	1	
t_{dly}	0.230	0.248	0.064	0.012	0.808	< 0.0001	1

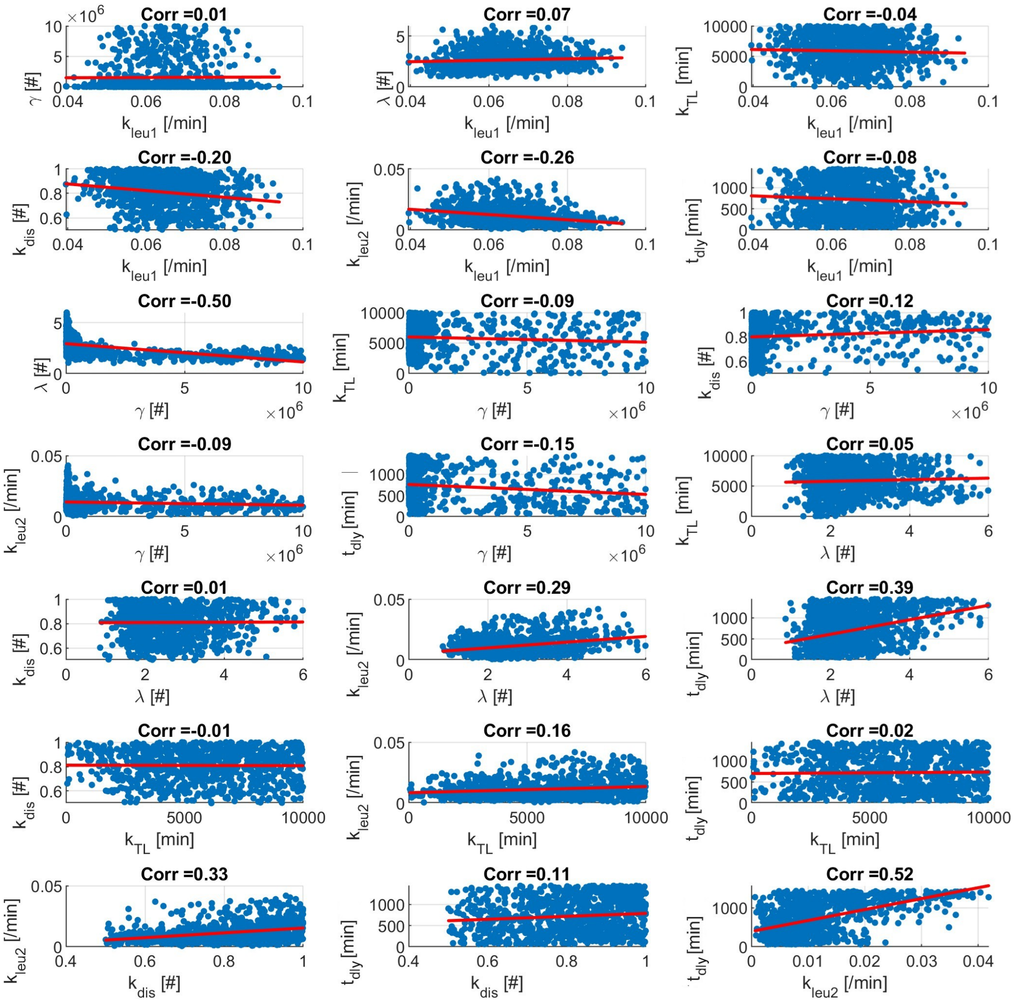

Fig S4. "Quadrants" method - Scatter plot of each pair of parameter estimates along with the the estimated linear regression curve (red lines). For each plot the Pearson correlation coefficient r is reported on the top.

Table S3. "Quadrants" method - P-values of the Pearson correlation coefficients.

	k_{leu1} [/min]	γ [#]	λ [#]	k_{TL} [min]	k_{dis} [#]	k_{leu2} [/min]	t_{dly} [min]
k_{leu1} [/min]	1						
γ [#]	0.815	1					
λ [#]	0.026	< 0.0001	1				
k_{TL} [min]	0.176	0.003	0.114	1			
k_{dis} [#]	< 0.0001	0.0001	0.805	0.791	1		
k_{leu2} [/min]	< 0.0001	0.005	< 0.0001	< 0.0001	< 0.0001	1	
t_{dly} [min]	0.017	< 0.0001	< 0.0001	0.537	0.0007	< 0.0001	1

Parameter Estimates Comparison Between “Bins” and “Quadrants” methods for “Experiment CA”

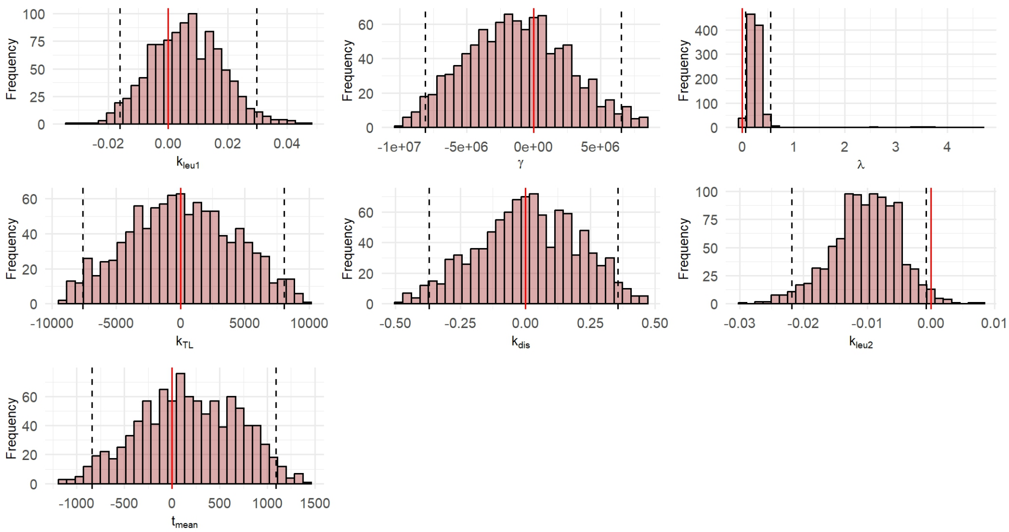

Fig S5. Posterior distributions of the differences $D_k = Q_k – B_k$. Each panel displays the posterior distribution of the difference (D_k) between parameter estimates obtained using the “Bins” (B_k) and “Quadrants” (Q_k) methods for a generic parameter k in “Experiment CA”. The dashed black lines indicate the limits of the 95% Credible Intervals, and the continuous red line marks the zero value. When Credible Intervals encompass zero the two methods do not yield significantly different parameter estimates.

Parameter Estimates Comparison Between ”Experiment CC” and ”Experiment CA” Using the “Bins” method

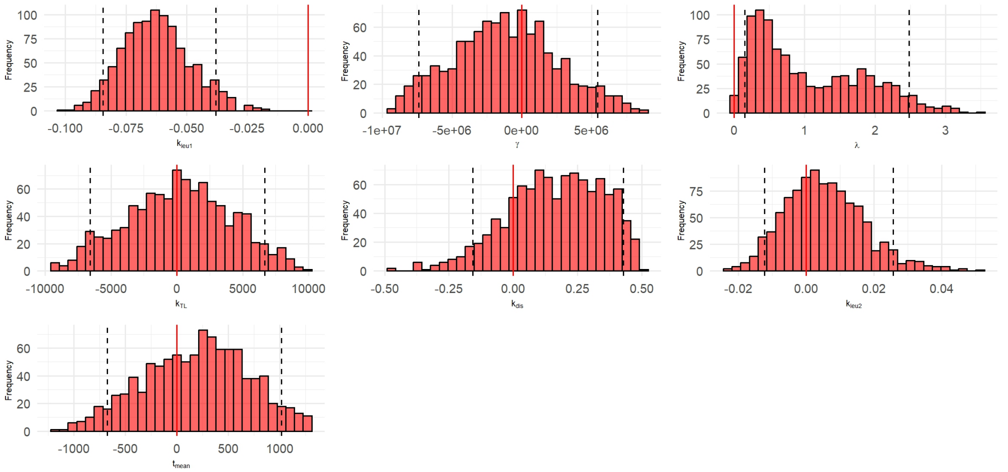

Fig S6. Posterior distributions of the differences $D_k = CC^B_k – CA^B_k$. Each panel displays the posterior distribution of the difference (D_k) between parameter estimates obtained in the “Experiment CC” (CC^B_k) and in the “Experiment CA” (CA^B_k) with the “Bins” method for a generic parameter k. The dashed black lines indicate the limits of the 95% Credible Intervals, and the continuous red line marks the zero value.

Parameter Estimates Comparison Between "Experiment CC" and "Experiment CA" Using the "Quadrants" method

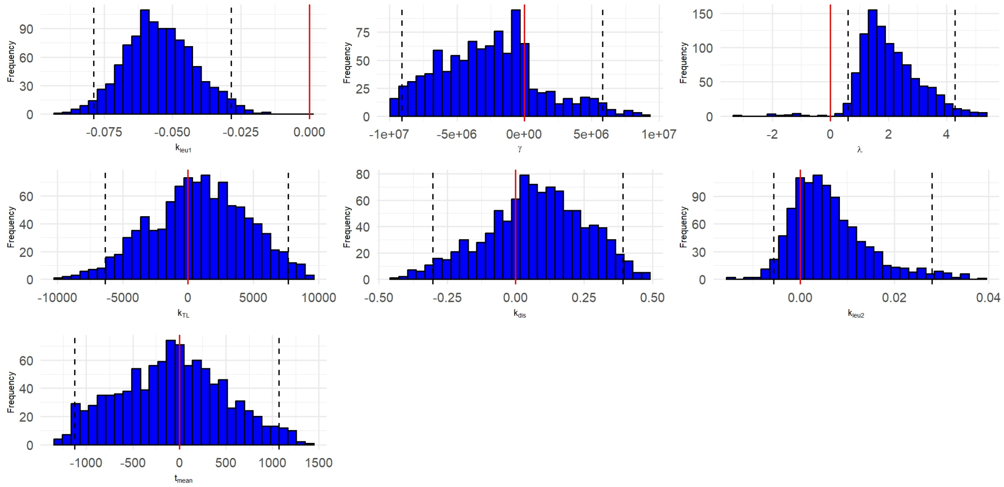

Fig S7. Posterior distributions of the differences $D_k = CC_k^Q – CA_k^Q$. Each panel displays the posterior distribution of the difference (D_k) between parameter estimates obtained in the "Experiment CC" (CC_k^Q) and in the "Experiment CA" (CA_k^Q) with the "Quadrants" method for a generic parameter k. The dashed black lines indicate the limits of the 95% Credible Intervals, and the continuous red line marks the zero value.

Sequential Importance Sampling (SIS) algorithm

Let us start by first providing a short overview of the Importance Sampling (IS) method, a sampling tool used for Monte Carlo computing, where a mathematical expectation with respect to a target distribution is approximated by a weighted average of random samples drawn from another distribution.

Let π be the probability distribution of a random variable X we want to sample from (that is the target distribution) and let $E_\pi[f(X)]$ the expectation of $f(X)$:

$$E_\pi[f(X)] = \int f(x)\pi(x)dx \tag{1}$$

Then for any probability density $q(x)$ that satisfies $q(x) > 0$ whenever $f(x)\pi(x) \neq 0$, one has

$$E_\pi[f(X)] = E_q[\omega(x)f(X)]. \tag{2}$$

where $\omega(x) = \frac{p(x)}{q(x)}$ is the importance weight and $E_q[\cdot]$ denotes the expectation with respect to $q(x)$ so that

$$E_q[\omega(x)f(X)] = \int \omega(x)f(x)q(x)dx. \tag{3}$$

A sample of independent draws $x^1, ..., x^m$ from $q(x)$ can be therefore used to estimate $E_\pi[f(X)]$ via the Monte Carlo approximation:

$$\hat{E_\pi}[f(X)] = \frac{1}{m}\sum_{j=1}^{m} w(x^j)f(x^j) = \frac{1}{m}\sum_{j=1}^{m} \frac{\pi(x^j)}{q(x^j)}f(x^j). \tag{4}$$

Therefore, when little is known about the structural properties of the target density $\pi(x)$ and it is impossible or difficult to sample directly from it, one can identify a distribution $q(x)$ that is easy to sample from and yet provides a good approximation to $\pi(x)$ by using importance sampling weights.

The Sequential Importance Sampling (SIS) is a variant of the IS designed for sequential estimation problems. It extends the IS algorithm by recursively sampling and updating samples at each time step, making it particularly useful for dynamic systems where the target distribution changes over time.

In SIS therefore, one reaches the target distribution π_T through a series of intermediate distributions, π_t, $t = 1, ..., T-1$. If it is hard to sample from these distributions, one can use the idea of importance sampling described above to sample from a series of proposal distributions q_t and weight the obtained samples by importance weights

$$\omega_t(x_t) = \frac{\pi_t(x_t)}{q_t(x_t)}. \tag{5}$$

At time t=1, we start with a target distribution π_1 and the proposal distribution can be in the simplest case $q_1 = \pi_1$. Then at time t=2 the new target distribution is π_2. To build the associated IS distribution q_2 we use particles sampled at time t=1 $\{X_1^{(i)}\}$. If π_1 and π_2 are not too different, then it should be possible to move the particles $\{X_1^{(i)}\}$ in the region at high probability density of π_2 [1].

In general at time $t-1$ we have N particles $\{X_{t-1}^{(i)}\}$ which are distributed according to q_{t-1} and the objective is to move these particles by using a Markov kernel κ_t with density $\kappa_t(x_{t-1}, x_t)$ so that the particles $\{X_t^{(i)}\}$ are marginally distributed according to:

$$q_t(x_t) = \int q_{t-1}(x_{t-1})\kappa_t(x_{t-1}, x_t)dx_{t-1} \tag{6}$$

where q_{t-1} is the previous proposal distribution and κ_t is a Markov kernel.
The SIS algorithm then works as it follows:

- set t=1 and for $i = 1, ..., N$, draw $X_1^{(i)} \sim q_1$, compute $\omega_1(X_1^{(i)})$ from (5) and normalize.
- set $t = t+1$, if $t = T+1$, stop, otherwise for $i = 1, ..., N$ draw $X_t \sim \kappa_t(X_{t-1}^{(i)}, \cdot)$. Evaluate $\omega_t(X_t^{(i)})$ from (5) with $q_t(x_t)$ computed from the Monte Carlo approximation of 6 and normalize.

The ABC-SMC is a special case of the SIS algorithm and its derivation is detailed in [2]

References

1. Del Moral P, Doucet A, Jasra A. Sequential monte carlo samplers. Journal of the Royal Statistical Society Series B: Statistical Methodology. 2006;68(3):411–436.

2. Toni T, Welch D, Strelkowa N, Ipsen A, Stumpf MP. Approximate Bayesian computation scheme for parameter inference and model selection in dynamical systems. Journal of the Royal Society Interface. 2009;6(31):187–202.
